# Proximal femoral head transcriptome reveals novel candidate genes related to epiphysiolysis in broiler chickens

**DOI:** 10.1186/s12864-019-6411-9

**Published:** 2019-12-30

**Authors:** Jane de Oliveira Peixoto, Igor Ricardo Savoldi, Adriana Mércia Guaratini Ibelli, Maurício Egídio Cantão, Fátima Regina Ferreira Jaenisch, Poliana Fernanda Giachetto, Matthew Lee Settles, Ricardo Zanella, Jorge Augusto Petroli Marchesi, José Rodrigo Pandolfi, Luiz Lehmann Coutinho, Mônica Corrêa Ledur

**Affiliations:** 1 Embrapa Suínos e Aves, Rodovia BR-153, Km 110, Distrito de Tamanduá, Caixa Postal: 321, Concórdia, Santa Catarina 89715-899 Brazil; 2 Programa de Pós-Graduação em Ciências Veterinárias, Universidade Estadual do Centro-Oeste, Guarapuava, Paraná, Brazil; 3 Universidade do Contestado, Concórdia, Santa Catarina Brazil; 4 Programa de Pós-Graduação em Zootecnia, UDESC-Oeste, Chapecó, SC Brazil; 5 Embrapa Informática Agropecuária, Campinas, SP Brazil; 6 UC Davis Genome Center, University of California, Davis, CA USA; 7 Universidade de Passo Fundo, Passo Fundo, RS Brazil; 8 Programa de Mestrado em BioExperimentação, UPF, Passo Fundo, RS Brazil; 9 Department of Genetics, Ribeirão Preto Medical School, University of São Paulo, Ribeirão Preto, SP Brazil; 10 Universidade de São Paulo, Piracicaba, SP Brazil

**Keywords:** BCO, Femoral head necrosis, Femur growth plate, RNA-sequencing, Leg problems

## Abstract

**Background:**

The proximal femoral head separation (FHS) or epiphysiolysis is a prevalent disorder affecting the chicken femur epiphysis, being considered a risk factor to infection which can cause bacterial chondronecrosis with osteomyelitis in broilers. To identify the genetic mechanisms involved in epiphysiolysis, differentially expressed (DE) genes in the femur of normal and FHS-affected broilers were identified using RNA-Seq technology. Femoral growth plate (GP) samples from 35-day-old commercial male broilers were collected from 4 healthy and 4 FHS-affected broilers. Sequencing was performed using an Illumina paired-end protocol. Differentially expressed genes were obtained using the edgeR package based on the False Discovery Rate (FDR < 0.05).

**Results:**

Approximately 16 million reads/sample were generated with 2 × 100 bp paired-end reads. After data quality control, approximately 12 million reads/sample were mapped to the reference chicken genome (Galgal5). A total of 12,645 genes were expressed in the femur GP. Out of those, 314 were DE between groups, being 154 upregulated and 160 downregulated in FHS-affected broilers. In the functional analyses, several biological processes (BP) were overrepresented. Among them, those related to cell adhesion, extracellular matrix (ECM), bone development, blood circulation and lipid metabolism, which are more related to chicken growth, are possibly involved with the onset of FHS. On the other hand, BP associated to apoptosis or cell death and immune response, which were also found in our study, could be related to the consequence of the FHS.

**Conclusions:**

Genes with potential role in the epiphysiolysis were identified through the femur head transcriptome analysis, providing a better understanding of the mechanisms that regulate bone development in fast-growing chickens. In this study, we highlighted the importance of cell adhesion and extracellular matrix related genes in triggering FHS. Furthermore, we have shown new insights on the involvement of lipidemia and immune response/inflammation with FHS in broilers. Understanding the changes in the GP transcriptome might support breeding strategies to address poultry robustness and to obtain more resilient broilers.

## Background

The poultry meat industry is extraordinarily efficient in supplying high-quality and affordable food for human and animal consumption. Chicken is one of the main sources of animal protein for humans, and nowadays is the second most widely consumed meat in the world [1]. In the last decades, the improvement in poultry genetic potential was remarkable, and was the major factor for rapid growth and feed efficiency [2, 3]. As a consequence, the modern broilers have experienced huge phenotypic and genetic changes. Chicken production efficiency contributes to reducing environmental impact, as demonstrated by the smaller water footprint compared with other livestock production [4]. However, the genetic progress in production traits was concomitant with the appearance of undesirable effects, including increased skeletal defects, metabolic disorders and altered immune function [5].

Zuidhof et al. [3] showed that broiler growth rate has increased by over 400% with a concomitant 50% reduction in feed conversion ratio from 1957 to 2005, when comparing representative lines of those years in identical environments. The metabolism has increased in meat-type chickens due to the intense selection for growth rate and the intensification of the production system; as a consequence, metabolic disorders became a worldwide concern, causing more economic losses than infectious agents [6]. These conditions affect primarily the cardiovascular and musculoskeletal systems [6] and the incidence of bone problems is nowadays considered one of the main concerns to the poultry industry due to the significant economic losses and by severely compromising poultry welfare [6, 7]. Moreover, leg disorders are one of the major welfare problems in the poultry production system [8]. Severe leg disorders may compromise the broilers welfare due to pain and inability to walk, leading to feed and water restriction [7]. In addition, locomotor disorders violates four of the five freedoms that farmed animals are expected to enjoy according to the Farm Animal Welfare Council [9]. These problems have a high prevalence in the farms, ranging from 9 to 33% [10, 11]. The proximal femoral head is highly exposed to injury under strenuous conditions, especially in broilers. Femur and tibia growth plates are sites of huge mechanical stress [12] and the increase in body weight significantly alters the femur center of gravity [13].

A common disorder affecting the proximal femur epiphysis is the proximal femoral head separation or epiphyseolysis (FHS), which is the separation of the growth plate (GP) from the articular cartilage (AC) resulting in disarticulation of the coxofemoral joint [13, 14]. Durairaj et al. [15] consider FHS as a metabolic skeletal problem in fast-growing chickens that increases the femoral epiphysis vulnerability to separation and is implicated in subsequent problems, such as bacterial infection [6]. The real incidence of FHS in broiler chickens is difficult to estimate because the lesion remains subclinical and the etiologic basis of FHS in young poultry is not completely understood [13].

The FHS is a predisposing factor for infection and osteomyelitis [15], which are conditions related to the Bacterial Chondronecrosis with Osteomyelitis (BCO), also known as Femoral Head Necrosis (FHN) [16]. The BCO is considered the most important leg disorder and the main cause of lameness in commercial broiler flocks, mainly affecting heavy birds after 35 days of age [7, 12, 16, 17]. In BCO affected flocks, morbidity is around 15%, which results in significant losses [16, 18]. Therefore, in order to investigate the molecular events taking place during the onset and development of the epiphysiolysis in fast-growing chickens, the present study used the RNA-Seq technology to identify differentially expressed genes in the proximal femur head of normal and FHS-affected broilers.

## Results

### Transcriptome analysis

**Fig. 1:**
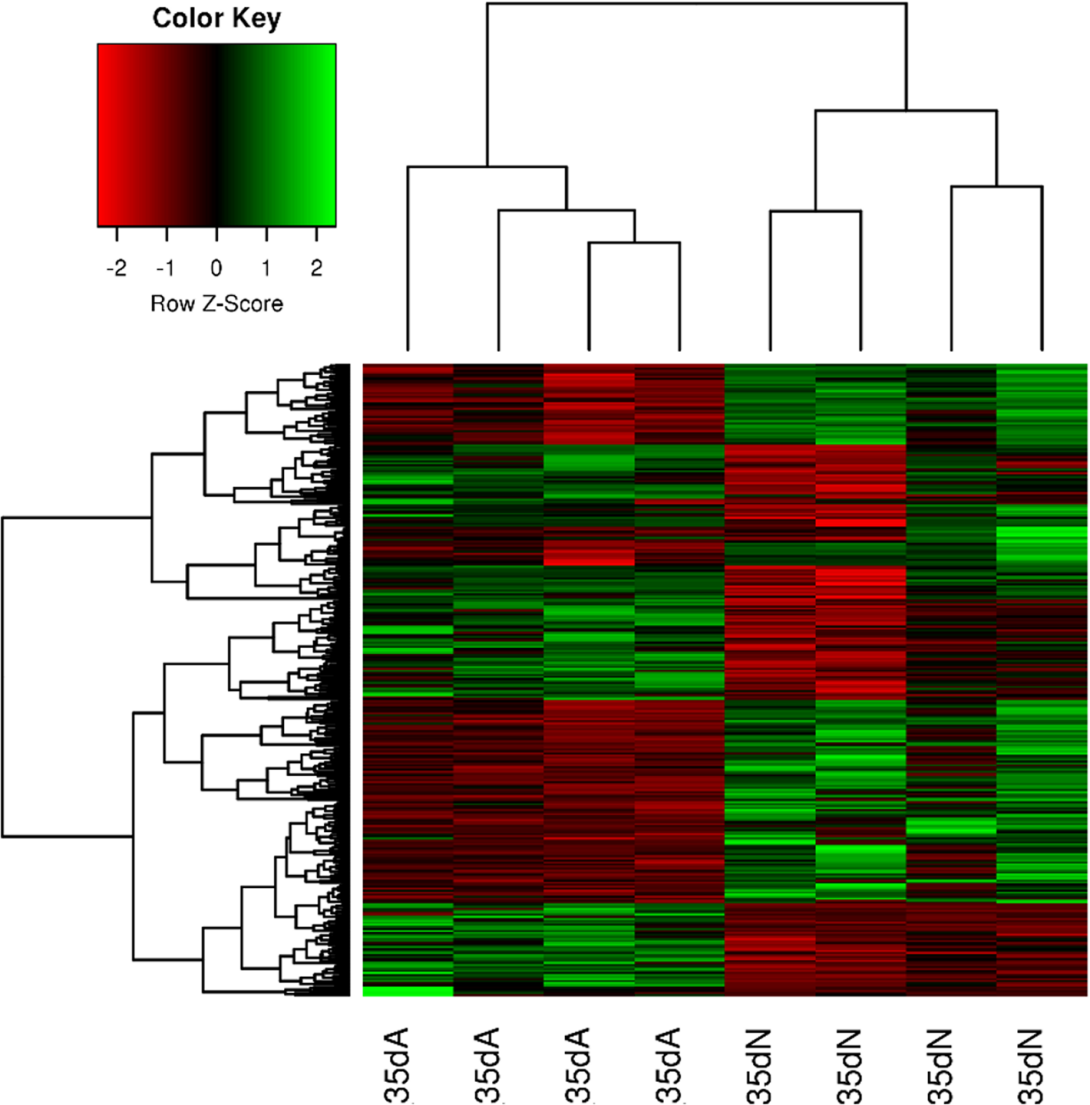
Gene cluster classification for FHS-affected and unaffected samples, with the 314 DE genes. In the heatmap, the expression for each gene is shown in the rows and samples are visualized in the columns, showing a hierarchical clustering of genes and samples. Genes are upregulated (in red) and downregulated (in green) in the affected samples

High-throughput RNA sequencing was used to generate a whole characterization of the proximal femur GP transcriptome of health and FHS-affected broilers. The multi-dimensional scaling (MDS) plot provided a spatial representation of data (Additional file 1) grouping samples in distinct clusters. It was possible to observe the homogeneity between samples from each group, which was also confirmed in the Heatmap plot (Fig. 1). The cluster analysis with the DE genes revealed a clear separation between FHS-affected and unaffected samples, suggesting homogeneity of the clinical classification of broilers in healthy and FHS-affected (Fig. 1).

Approximately, 16 million reads/sample were generated with 2 × 100 bp paired-end reads. After data quality control, in average, about 12 million reads/sample were mapped to the chicken reference genome (Galgal5, Ensembl release 87). The average reads mapped across samples was 98.99% and a total of 12,645 genes were expressed in the GP transcriptome. Out of those, 314 were DE between normal and FHS-affected broilers (FDR < 0.05), being 154 upregulated and 160 downregulated (Additional file 2) in the affected compared to normal group. The top 10 down and upregulated transcripts are shown in Table 1.

### Functional analyses

**Fig. 2:**
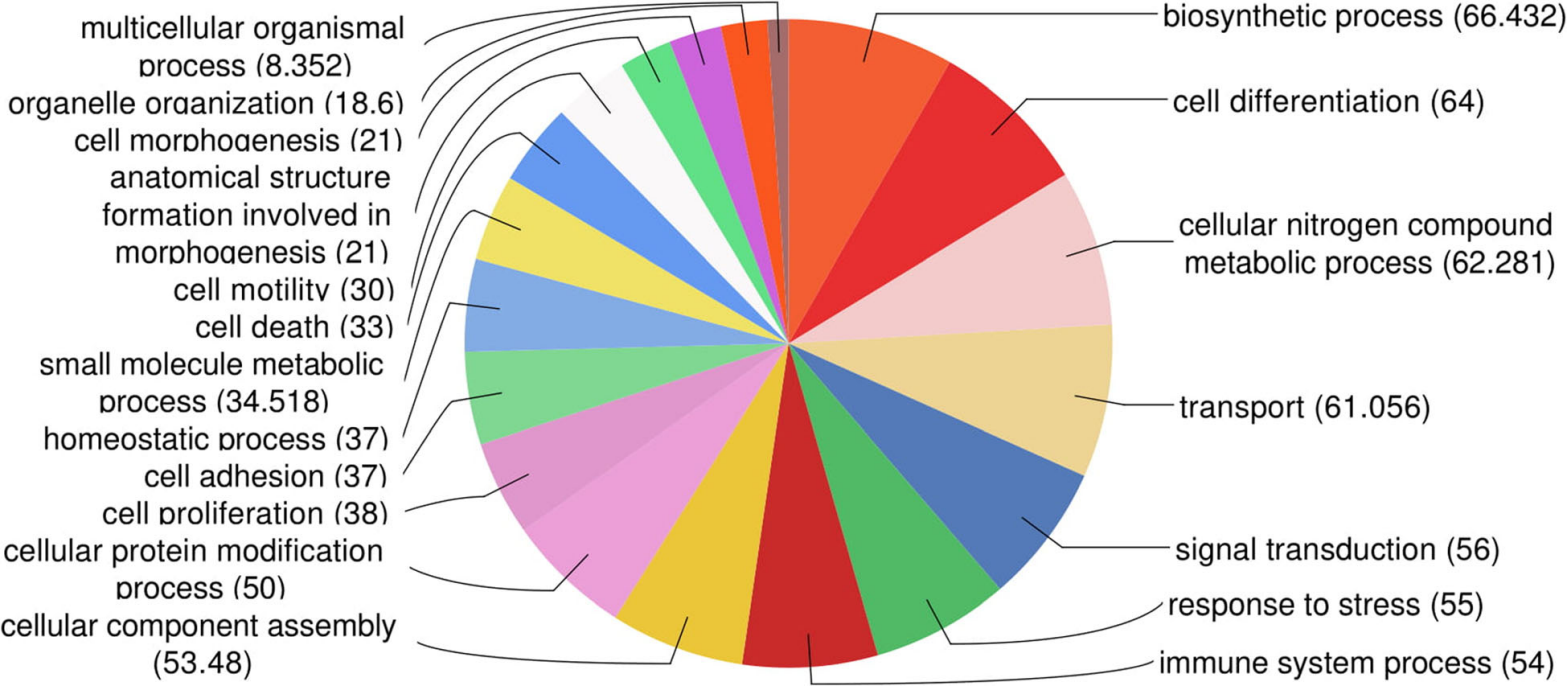
Distribution of DE genes according to the gene ontology category of biological processes in Blast2GO analysis

**Fig. 3:**
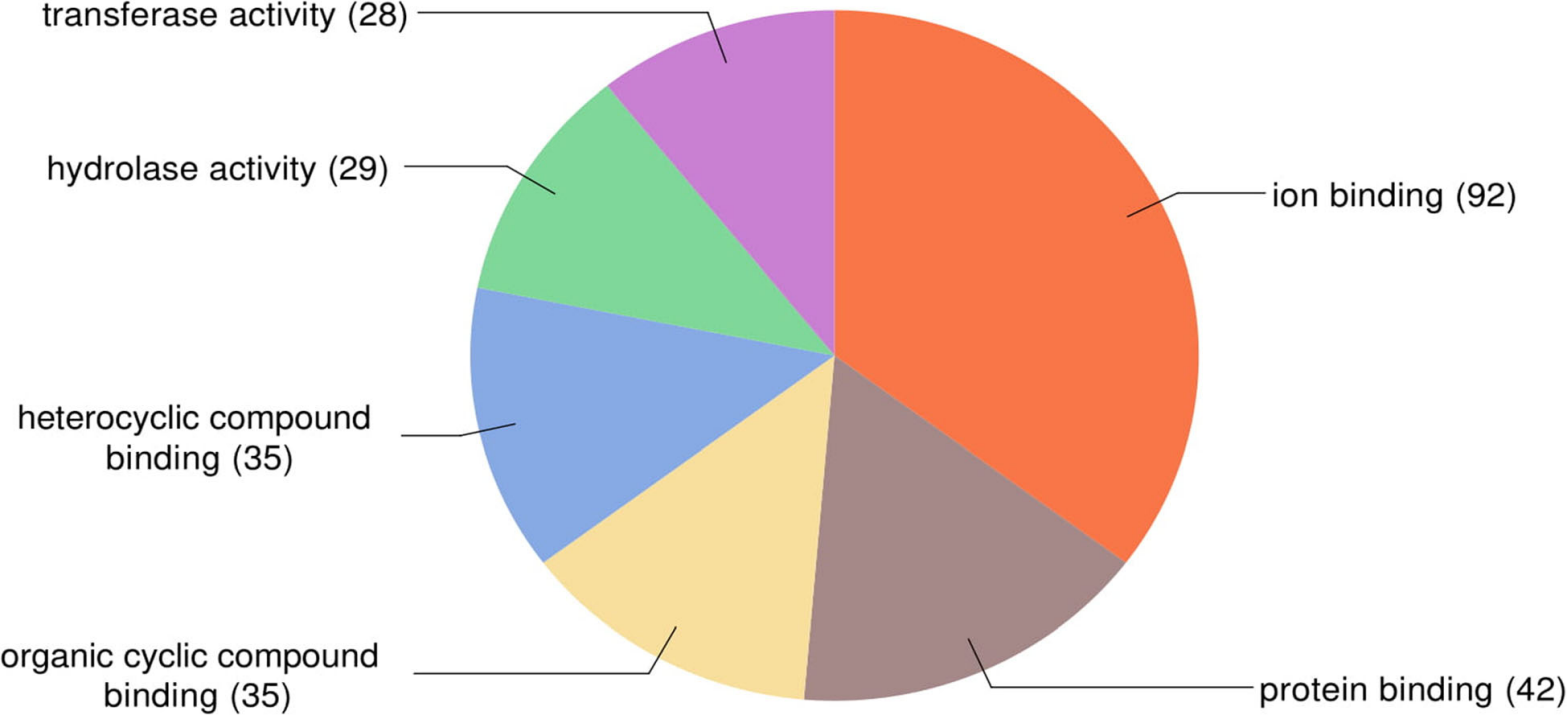
Distribution of DE genes according to the gene ontology category of molecular function in Blast2GO analysis

The gene ontology analysis performed with the DE genes in Blast2GO showed that 290 annotated genes were enriched in 19 biological process (Fig. 2). According to the GO molecular function (Fig. 3), most of the DE genes presented binding functions, such as binding to ions, protein, heterocyclic compound and organic cyclic compound. Furthermore, transferase and hydrolase activity were also well represented molecular functions of the DE genes in the growth plate tissue (Fig. 3).

**Fig. 4:**
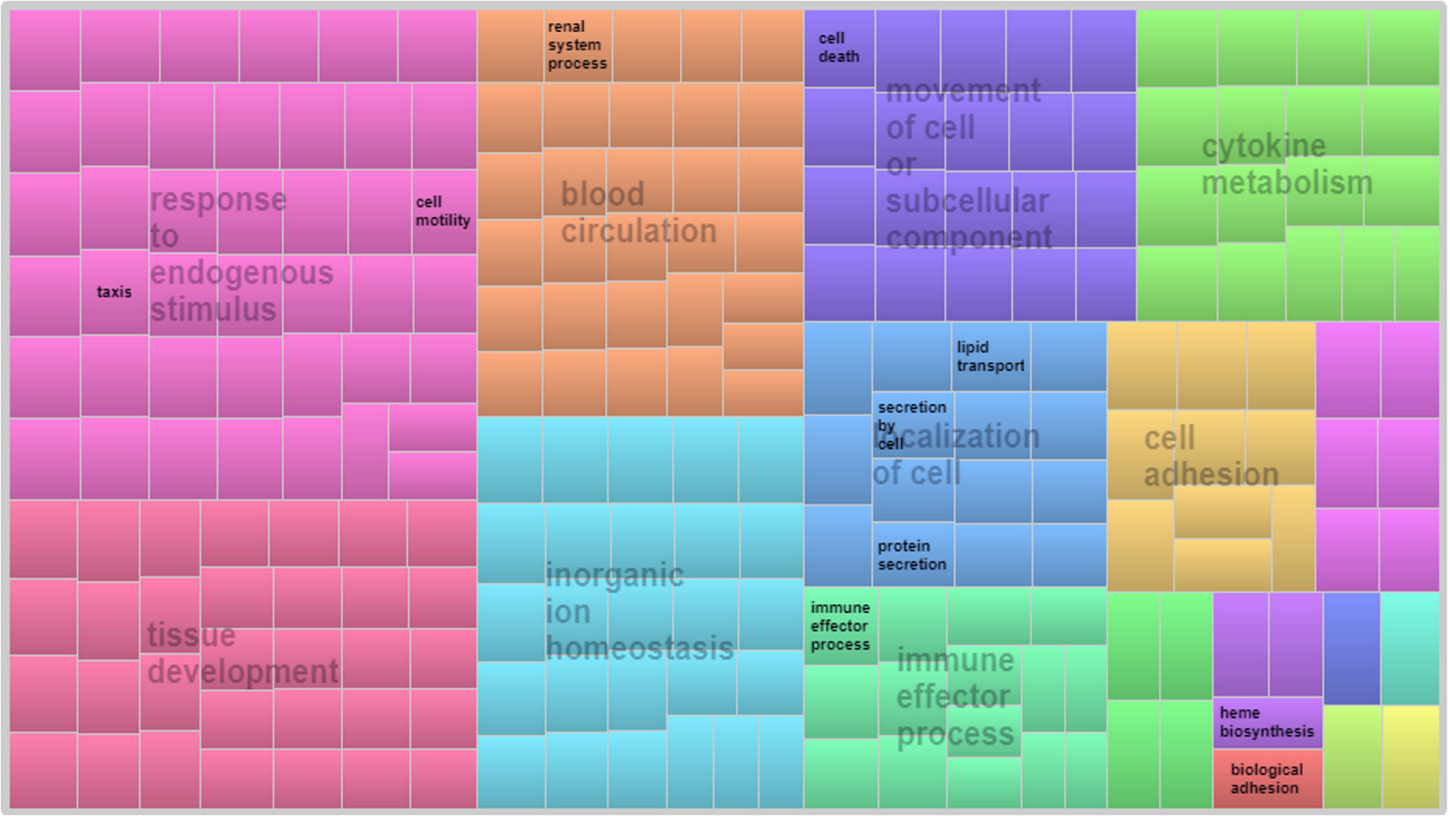
Superclusters of biological process enriched for up and downregulated genes related to FHS. Different colors show different superclusters and the size of each box is determined by the uniqueness of the categories

Gene ontology analysis was also performed in DAVID database, finding a total of 226 BP (Additional file 3), which were summarized in 9 superclusters with the REViGO tool (Fig. 4), including the following BP: response do endogenous stimulus, tissue development, blood circulation, inorganic ion homeostasis, immune effector process, cytokine metabolism and cell adhesion.

**Fig. 5:**
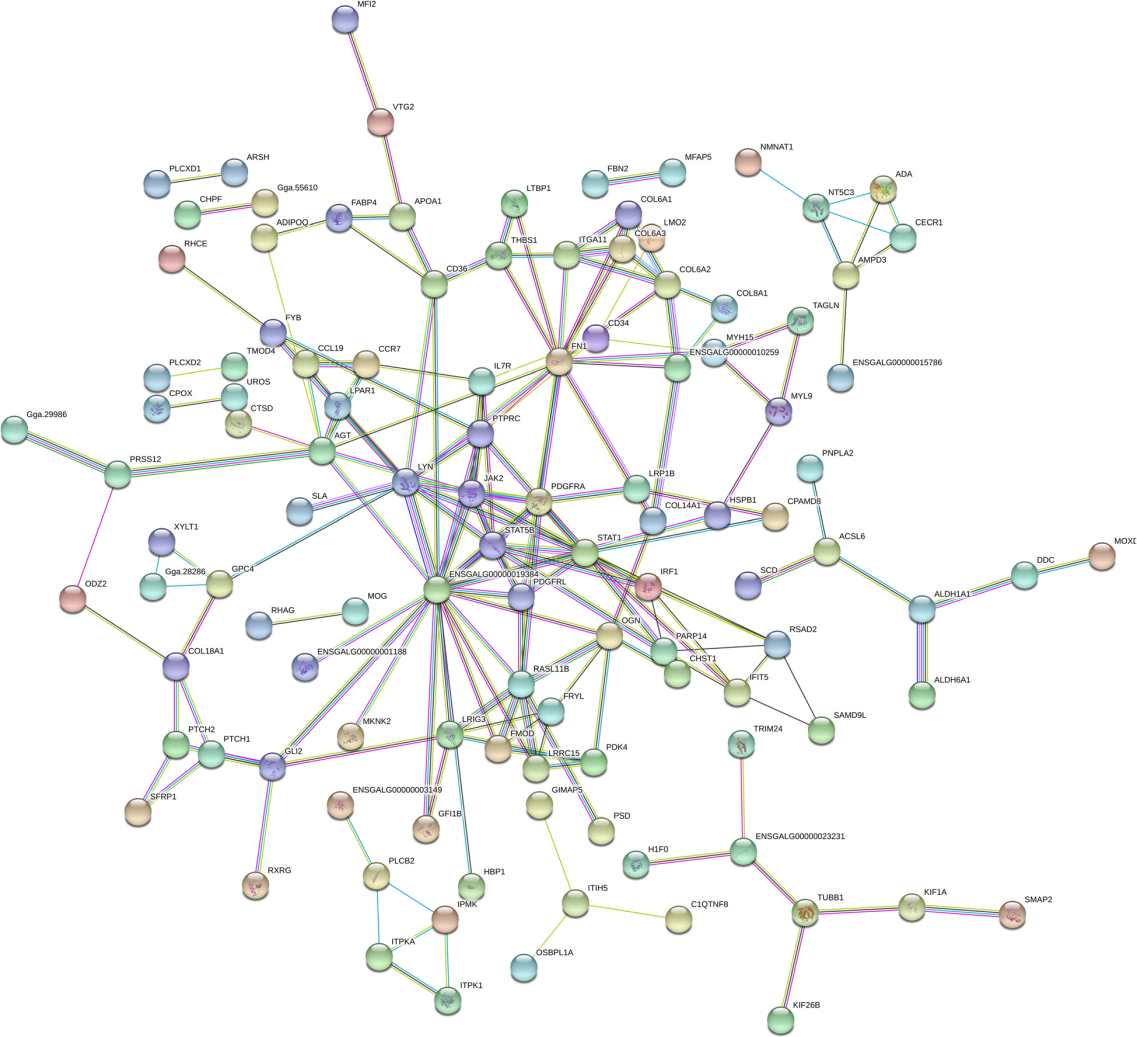
Gene network of differentially expressed genes related to femoral head separation in broilers using Stringdb. Nodes indicate the number of predicted gene interactions

**Fig. 6:**
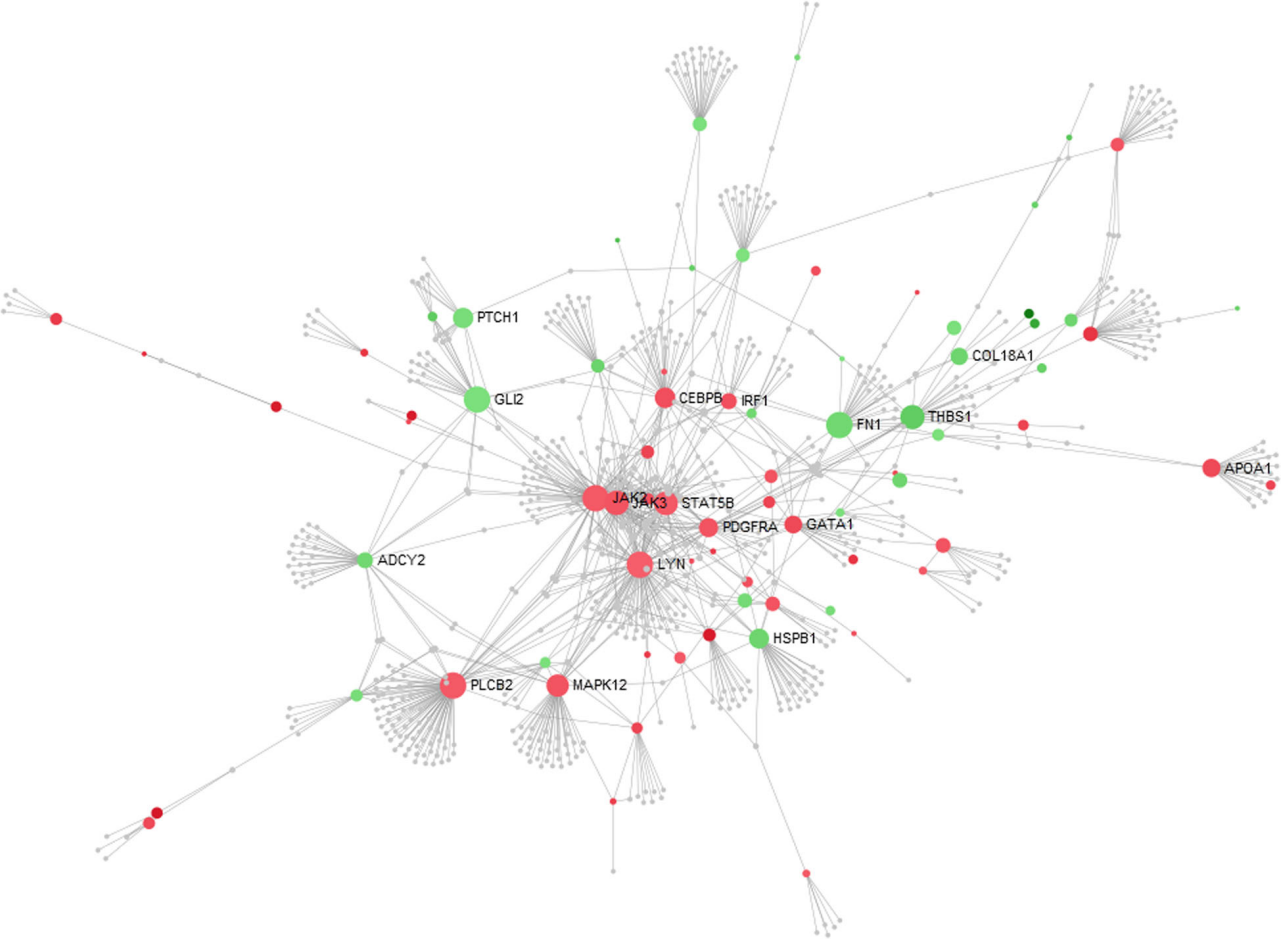
Gene enrichment analysis of differentially expressed genes and biological processes related to femoral head separation in broilers using the NetworkAnalyst tool. Nodes indicate the number of predicted gene interactions. Strong and large circles contain high number of genes. Red circles are the upregulated and green circles are the downregulated DE genes in FHS-affected compared to normal broilers

A strong evidence of interaction of several DE genes was observed in the gene network analysis (Fig. 5). A central group of genes was found, such as *JAK2*, *PDGFRA, STAT5A, PDGFRL* and *MAPK12* (ENSGALG00000019384) that are mostly involved in blood circulation and angiogenesis (Fig. 5). These genes were also connected with many genes related to bone differentiation (i.e. *OGN, ADIPOQ, GLI2),* extracellular matrix *(*i.e. *collagen genes, FN1),* tissue homeostasis *(*i.e. *JAK2, IL7R, RHAG, EPB42)* and immune response *(*i.e. *IL7R, IRF1, JAK2)* (Fig. 5)*.* Other 5 small branch of genes were found, with no direct node with the main group of genes, being: 1); *PLCB2, ITPKA, ITPK1* and *ITPR3;* 2) *OSBPL1A, ITIH5, C1QTNFB and GIMAP5;* 3) *H1F0, TRIM23, TUBB1, KIF26B, KIF1A and SMAP2;* 4) *PNPLA2, ACSL6, SCD, ALDH1A1, DDC* and *ALDH6A1* and 5) *NMNAT1, NT5C3, ADA, CECR1, AMPD3* and *BTBD10* (Fig. 5). Nevertheless, some of those genes could be considered hubs since they are involved in the expression of several other genes (Fig. 6).

**Fig. 7:**
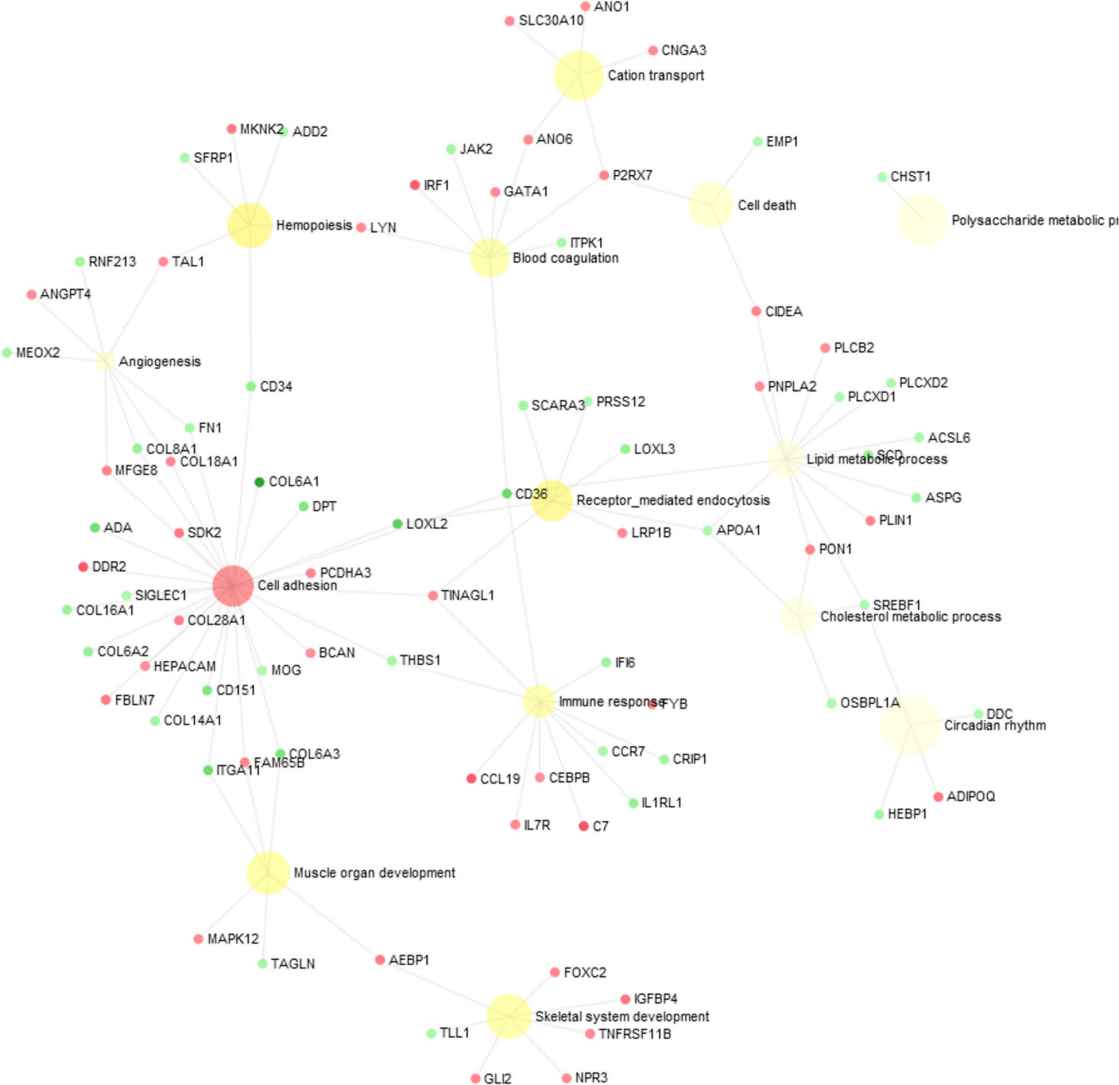
Bipartite gene network of differentially expressed genes related to femoral head separation in broilers using NetworkAnalyst tool. Nodes indicate the number of predicted gene interactions. Red circles are the upregulated and green circles are the downregulated DE genes in FHS-affected compared to normal broilers. The hub genes are highlighted in green and red

It is possible to observe that cell adhesion is one of the most enriched BP, grouping genes that interact with angiogenesis, muscle development, skeletal systems, immune response and lipid metabolism (Fig. 7).

### Validation by quantitative PCR

**Fig. 8:**
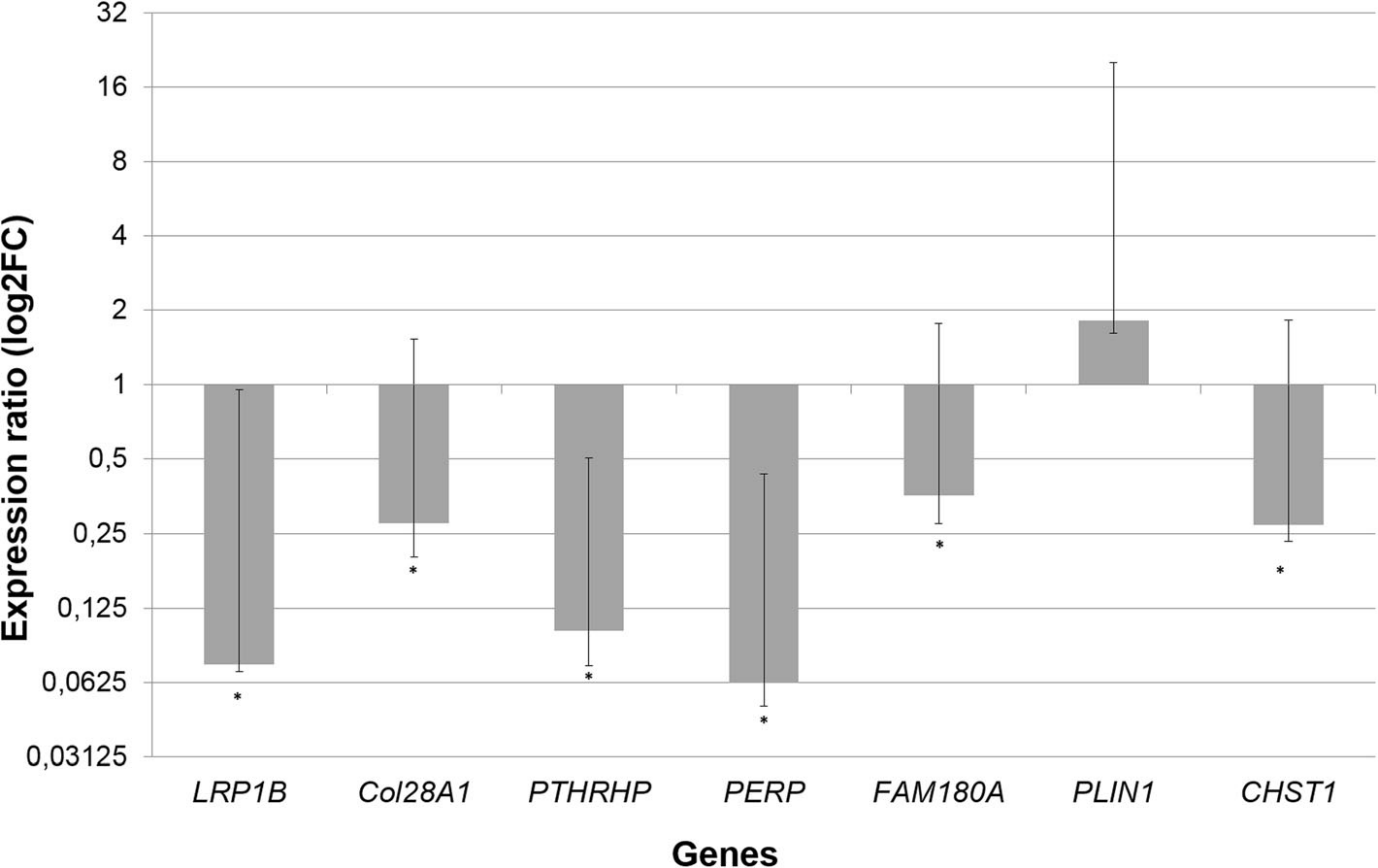
Ratio of gene expression in femur between normal and FHS-affected broilers, normalized with the *RPL4* and *RPL30* reference genes (*p < 0.05)

In the qPCR analysis, five of the six analyzed genes (*LRP1B, Col28A, PERP2, FAM180A* and *CHST1*) were differentially expressed (*P* < 0.05) in the proximal femur GP between normal and FHS-affected groups. Furthermore, all of these genes kept the same expression profile observed in the RNA-Seq analysis (Table 2, Fig. 8), confirming the results from our RNA-Seq study. Although the *PLIN1* was not DE between groups in the qPCR analysis, it had similar expression levels between the two studies, following the same pattern of upregulation in FHS-affected broilers. The difference between the statistical significance from the two approaches could be due to the heterogeneity of the expression among samples within groups.

## Discussion

The selection of broiler chickens for rapid growth and increased muscle yield has resulted in some undesirable consequences including a high incidence of skeletal problems [15]. Separation of the femoral epiphysis from the articular cartilage (FHS) may be a metabolic problem that leads the femur vulnerable to other anomalies. The endochondral ossification of long bones consists of the proliferation and differentiation of chondrocytes in the epiphysial growth plate [19]. Although the ossification is a conserved process across several mammal species, chickens present some differences in bone physiology, including fast bone remodeling [20]. In the growing phase, femur and tibia have a very fast development [21], and they continue to grow until the adult age. This pattern has been observed by several authors studying the rapid leg bone growth in broilers [22–24]. Moreover, it has been a consensus that the skeleton does not mature fast enough to support the growth potential of modern broilers [18, 25] and that femur and tibia growth plates are sites of huge mechanical stress, especially due to the high growth rates [12]. In this sense, the increased metabolism and mechanical stressful condition are predisposing factors to some of the most important leg problems in the femur and tibia growth plates, such as FHS, BCO and tibial dyschondroplasia (TD). Therefore, the global transcriptome analyses of the femur growth plate tissue in the proximal head can unravel regulatory pathways that control the development of skeletal abnormalities related to FHS in chickens.

Here, we provided the first FHS-related transcriptome and identified a group of genes involved with FHS in broilers. These findings are an important resource of functional candidate genes for future studies on bone development in fast-growing chickens. The main BP potentially involved in triggering FHS were those related to chicken growth, such as bone development, extracellular matrix organization, lipid metabolism, blood circulation and cell adhesion (Figs. 5 and 6). On the other hand, BP associated to apoptosis or cell death and immune response processes, also found in our study, could be a consequence of the disruption on growth (Additional file 3, Fig. 7). The main BP related to physiological mechanisms involved in FHS are discussed below.

### Bone development, extracellular matrix organization and cell adhesion

In broilers, 35 days of age is an important phase of the femur development during a commercial growing period [26]. At this age, the femur length as a function of body weight reaches a plateau, differing from the tibia bone that continues to lengthen [21]. Some authors indicate that most cases of BCO, considered a progression of FHS, develop after 35 days of age [12, 16, 17], possibly because the body weight increases faster than the femur development [21]. Furthermore, it has been observed that at early ages, 0 to 7 days, fast-growing broilers have higher mean volumetric bone mineral density and heavier and longer bone than medium-growing chickens, which could indicate that bone development is adequate in the early life or even during embryogenesis in fast-growing broilers [26].

The epiphysiolysis has been classified as a disorder related to the growing phase and a large number of DE genes in this study were associated to BP related to skeletal system development, ossification, osteoblast differentiation, cell development, and others (Additional file 3, Fig. 7). These BP are directly correlated with bone function, and several genes, such as *ACSL6, ADIPOQ, BNC2, LOXL2, COL6A1, COL8A1, CYTL1, FAM65B, FN1, GLI2, CD36, ITGA1* and *OGN* showed reduced expression in the FHS-affected broilers (Additional file 3, Fig. 5).

The bone extracellular matrix is a keystone in skeletal development and a precisely regulated expression of cellular and extracellular matrix genes is required to the correct osteogenesis [27]. The ECM is composed by a proteinaceous fiber component (collagen) and an amorphous ground substance (proteoglycan) being a dynamic network of molecules secreted by cells under regulation of transcription factors, signaling molecules, hormones and local growth factors. According to Velleman [27], although the composition of the ECM is known, the function of each macromolecule components and their interaction is poorly understood and the role of the bone ECM in skeletal development has been ignored by the poultry scientific community.

Cell adhesion related BP could have a key function in triggering FHS (Fig. 4, Fig. 7), since it was the main hub to connect all of the other processes that were involved with FHS (Fig. 7). The *Lysyl oxidase-like-2* (*LOXL2*) gene can be highlighted since it catalyzes the oxidative deamination of peptidyl lysine residues, promoting the formation of lysyl-derived crosslinks in collagens and elastin [28], genes that were also DE in our study. LOXL2-catalyzed crosslinks are essential post-translational modifications in the biosynthesis and maintenance of the ECM, contributing to the tensile strength and stability of tissues [29]. Therefore, the downregulation of *LOXL2* and the other genes could reduce the cell adhesion, which could facilitate the separation of cartilage and bone tissues. Furthermore, this gene is also connected with immune biological responses, blood coagulation and lipid metabolism functions (Fig. 7).

Many biological processes related to ECM and also cell adhesion were enriched, such as ECM organization, glycosaminoglycan metabolic process, aminoglycan metabolic process, extracellular structure organization, connective tissue development, biological adhesion, regulation of cell adhesion, cell adhesion, cell-matrix adhesion, and others (Additional file 3), where DE genes were downregulated in FHS-broilers (*ABI3BP, ADIPOQ, CD34, CD151, CHST1, COL6A1, COL6A3, COL8A1, COL16A1, CYTL1, FN1, GLI2, HSPB1, ITGA11, ITIH5, KIF26B, LOXL2, MFAP5, MYL9, PRRX1, TENM2, THBS1, TINAGL1,VIT* and *XYLT1*) (Figs. 5, 6 and 7). Here, an important downregulated gene to be discussed is the *CHST1,* which participates in the glycosaminoglycan (GAGs) and aminoglycan metabolic processes involved in the ECM metabolism. The *CHST1* (Carbohydrate Sulfotransferase 1) is a membrane bound sulfotransferase potentially involved in the production of L-selectin ligands, important for the recognition of various immunological receptors involved with the recruitment of macrophages to the tissue inflammation sites [30]. In addition, the GAGs can bind to many genes responsible for ossification and osteoblast formation, such as fibroblast growth factors (FGFs) and bone morphogenetic proteins (BMPs), regulating the skeletogenesis [31]. *CHST1* differential expression pattern between health and FHS-affected group was also confirmed by qPCR (Table 2). Moreover, using the same experimental population, Petry et al. [32] found that *Adiponectin* (*ADIPOQ*), *Paired related Homeobox 1* (*PRRX1*), *ABI family member 3 NESH binding protein P* (*ABI3B*) and *COL8A1* genes were also downregulated in FHS-affected group by qPCR, confirming their involvement with ECM, cell adhesion and bone formation.

According to the expression profile in this case-control study, a large number of coding genes for ECM structural proteins were downregulated, such as *FN1* (Fibronectin 1)*, FBLN7* (fibulin 7), *DPT* (dermatopontin), *GPC4* (glypican 4), *BCAN* (brevican), *FMOD* (fibromodulin), *FBN2* (fibrillin 2), *VIT* (vitrin), *CHPF* (chondroitin polymerizing factor), *SDK2* (sidekick cell adhesion molecule 2), *DBN1* (drebrin 1), *SDC3* (syndecan 3), *ITGA11* (integrin, alpha 11) and *OGN* (osteoglycin) (Fig. 5). Also, several members of the collagen family were downregulated in FHS-affected broilers: *COL6A1* (collagen, type VI, alpha 1), *COL6A2* (collagen, type VI, alpha 2), *COL6A3* (collagen, type VI, alpha 3), *COL8A1* (collagen, type VIII, alpha 1), *COL14A1* (collagen, type XIV, alpha 1), *COL18A1* (collagen type XVIII alpha 1 chain) and *COL28A1* (collagen, type XXVIII, alpha 1). Here, the *FN1* gene can be highlighted, because it is a multidomain glycoprotein with multiple adhesive properties binding cells and their extracellular matrices, and was recognized to be the target for a large number of bacteria [33]. The *FN1, Col8A1, Col18A1 and MFGE8* are also involved with angiogenesis (Fig. 7), and both *FN1* and *Col18A1* were downregulated in FHS-affected broilers, interacting with other BP (Fig. 6).

Moreover, most of the DE genes in our study presented binding molecular functions (MF), such as binding to ions, protein, heterocyclic compound and organic cyclic compound (Fig. 3). The molecular binding function is tightly coupled with the pathological condition evaluated in this study, since FHS is the separation of the growth plate from the articular cartilage [14]. The binding function is an intrinsic ability of the ECM macromolecule components and it is essential to the cell-matrix interactions. Several BP of cell adhesion related to binding MF were enriched in our study: biological adhesion, cell adhesion, regulation of cell adhesion, cell-matrix adhesion, cell-substrate adhesion, cell adhesion mediated by integrin, positive regulation of cell adhesion, cell-cell adhesion, single organismal cell-cell adhesion and single organism cell adhesion (Additional file 3, Fig. 7). Most of the genes grouped in these previous BP were downregulated in the FHS-affected broilers, indicating reduction in cell adhesion and binding function in the femur GP. Altogether, the results indicate that the downregulation of the ECM genes could negatively affect the ECM integrity predisposing to the separation of growth plate from articular cartilage in the femur epiphysis. Since cell adhesion and ECM related genes are connecting the main BP identified in this study, it is possible that the disruption in these processes could be the first steps to initiate FHS in chickens.

### Blood circulation and angiogenesis

Another observed supercluster in REViGO analysis (Fig. 4) is blood circulation that grouped important BP related to bone development: blood circulation, hemopoiesis, angiogenesis and circulatory system development (Additional file 3). Blood circulation is connected with other observed BP, such as immune response and inflammation. It is known that tissue damage leads to an increase in blood flow, capillary permeability and in the influx of immune cells to repair the tissue. In BCO lesion, affected blood vessels may be completely occluded by bacteria or may contain bacterial clumps surrounded by inflammatory cells and fibrin thrombi [16]. This group of blood circulation BP was enriched with upregulated genes, such as *ADA, AGT, CBS, CEBPB, EPB42, GFI1B, IL7R, IRF1, JAK2, NPR3, PDGFRA, RSAD2* and *RHAG,* and downregulated genes in the affected group, such as *ADIPOQ, COL8A1, FN1, GLI2, HSPB1, LOXL2, MEOX2, MFAP5, MYH15, PTCH1, PRRX1, SFRP1, SREBF1, THBS1* and *TNMD* (Fig. 6, Fig. 7). An interesting gene grouped in this BP is *LOXL2,* which has biological functions in the regulation of angiogenesis [34], being intrinsically related to the hypoxic state [35]. The bone development is tightly connected to the normal growth of blood vessels during endochondral formation [36, 37] and the vascularization is essential for physiological processes of the skeletal system. Blood vessels control bone growth and homeostasis by angiogenic and angiocrine signals mediating the transport of circulating cells, oxygen, nutrients and waste products [36]. Low levels of *LOXL2* gene, a member of hypoxia-induced LOX, could contribute to the lack of vascularization in the growth plate of the femur. This pattern has already been observed in studies with human and mice [35, 38]. This gene is also essential to the ECM formation, as described previously. Therefore, angiogenesis and vascularization seem to be secondary processes involved in FHS.

*Angiopoietin 4* (*ANGPT4*) was upregulated in broilers with FHS. The angiopoietins are proteins with important role in vascular development and angiogenesis. However, this gene was not grouped in any BP using DAVID. In this study, *Angiopoietin-like 5* (*ANGPTL5*) was one of the top 10 downregulated genes (Table 1) and Petry et al. [32] have confirmed the downregulation of *ANGPTL5* in FHS-affected broilers by qPCR. Angiopoietin-like proteins (ANGPTLs) are a family of proteins structurally similar to the angiopoietins, with functional roles in lipid metabolism, glucose metabolism, inflammation and hematopoiesis [39].

### Lipid metabolism

Some DE genes identified in our study were involved with lipid biological processes, such as: neutral lipid metabolic process, triglyceride metabolic process, lipid transport, lipid localization, regulation of lipid transport, cholesterol transport, fat cell differentiation and brown fat cell differentiation. The *ABCA8, ALDH6A1, APOA1, CEBPB, OSBPL1A, P2RX7, PNPLA2, AGT* and *PDK4* genes were upregulated in affected broilers while *SREBF1, SFRP1*, *PTCH1, THBS1* and *ADIPOQ* were downregulated (Additional file 2 and Additional file 3). Durairaj et al. [15] consider FHS as a metabolic skeletal problem in fast-growing chickens and observed elevation in blood lipid levels in broilers with FHS, suggesting that elevated lipid levels may be a risk factor for femoral head problems in chickens. Here, the *ADIPOQ* was downregulated in the femur of FHS-affected broilers and its expression profile was confirmed by qPCR [32]. According to DAVID results, *ADIPOQ* participates in 107 out of 226 BP mapped in this case-control study (Additional file 3), including bone development, cell adhesion, immune response and apoptosis, except homeostasis. In broilers, *ADIPOQ* acts in the metabolism of lipids and carbohydrates, being involved in adipogenesis [40]. Moreover, adiponectin has important role in regulating bone metabolism, inhibiting the osteoprotegerin expression in the osteoblasts and, also, by increasing the differentiation of osteoclasts [41]. Therefore, the *ADIPOQ* is an important candidate gene, since it has already been associated with metabolic disorders, including arthritis, osteonecrosis and obesity in humans [42–44]. Moreover, this gene acts in several BP possibly involved with the causes of FHS in broilers, as discussed in our study, including lipidemia.

Furthermore, adiponectin binds to specific receptors, *ADIPOR1* and *ADIPOR2*. The *ADIPOR1* is the major receptor that mediates the glucose and lipid metabolism-related effects of adiponectin on target cells [45]. Adiponectin gene may be associated with adipose tissue deposition in chickens [40, 46]. Cruz et al. [47] found a SNP (g 729 C > T) in the *ADIPOR1* gene associated with thickness of the femur, suggesting its influence on bone integrity in broilers.

An important candidate gene to FHS condition in broilers is the *purinergic receptor P2X7* (*P2RX7*), a member of the purinoceptors for ATP family [48]. This gene participated in 102 BP in DAVID analyses (Additional file 3) and was involved in the majority of the BP discussed in this study. Besides being essential in DNA and RNA synthesis, purines are important components of several biomolecules associated to the energetic metabolism such as ATP, GTP, cAMP and NADH. In this study, two purine BP were found: purine nucleotide salvage, including genes *AMPD3* and *ADA*, and purine-containing compound salvage, including genes *CECR1, AMPD3* and *ADA*. Here, *adenosine monophosphate deaminase 3* (*AMPD3*) and *adenosine deaminase* (*ADA*) were upregulated in FHS-affected broilers and the *CECR1*, also named *adenosine deaminase* (*ADA2*), was downregulated. Also included in the purinergic metabolism is the *CHAT2* gene, the most upregulated gene in the FHS-affected broiler (Table 1).

In this transcriptome study, the *G0/G1 switch gene 2* (*G0S2*), a candidate gene for metabolic disorders, was upregulated in broilers with FHS. This gene has been considered a potent endogenous inhibitor of *adipose triglyceride lipase* (*ATGL)* revealing a unique mechanism governing lipolysis and fatty acid availability [49]. Zhang and coworkers [44] evidenced that the *G0S2* acts as a master regulator of tissue-specific balance of triglycerides storage, mobilization, partitioning of metabolic fuels between adipose and liver, and of the whole-body adaptive energy response.

About 64% of the genes grouped in BP related to lipid transportation and metabolism were upregulated, such as *APOA1, PNPLA2, ABCA8, P2RX7, OSBPL1A, ALDH6A1, CEBPB, AGT* and *PDK4.* Some of them also interact with genes involved in the ossification and inflammation (Fig. 5, Fig. 6). The lipids play a role in bone metabolism, since they can affect the function of bone cells through many pathways [50], such as the vitamin D and K absorption, regulating the mineral maturation and also affecting the bone mineral density [50]. Those lipids can be driven to the bone via circulation, but could also be produced by osteoblasts [51]. Therefore, accumulation of lipids in the bone cells might lead to an inflammatory environment, generating oxidative stress, which could inhibit the expression of bone differentiation markers [52]. Thus, the unbalance of the lipid metabolism could precede the cell adhesion and ECM bioprocesses on the onset of FHS in fast-growing broilers.

### Immune response genes

Immune biological processes were overrepresented in both Blast2GO and REViGO. The profile of DE genes observed in this study showed a global activation of the immune system, comprehending innate immunity genes and genes associated with the inflammatory response. Some of the main BP found in DAVID were: immune system process, immune system development, regulation of immune system process, T cell mediated immunity, leukocyte differentiation, immune response, response to cytokine, leukocyte activation, leukocyte proliferation, lymphocyte activation, inflammatory response, defense response, regulation of defense response, defense response to other organism and defense response to bacterium (Additional file 3). Among the enriched genes, *ADA, ALOX5AP, AMPD3, APOA1, AVD, C7, CEBPB, EPB42, FYB, GFI1B, IL7R, IRF1, JAK2, NLRC5, NPR3, PTPRC, PDK4, P2RX7, PDGFRA, RHAG* and *RSAD2* were upregulated in the epiphysiolysis group, while *ADIPOQ, CD151, CHGA, FN1, HSPB1, MFAP5, SFRP1, TINAGL1* and *THBS1* were downregulated. The upregulated genes were predominant in all 20 immune BP.

Several response BP were observed, including defense response to other organism enriched with genes *NLRC5, P2RX7, CHGA, CEBPB, AVD, PDK4, IRF1* and *RSAD2*, and defense response to bacterium enriched with *P2RX7, CHGA, CEBPB* and *AVD* genes. In both BP, only *CHGA* was downregulated in affected chickens. Although FHS may precede bacterial infection, these two BP are indicative of the presence of microorganisms in the GP tissue. The *P2RX7* receptor serves as a pattern recognition receptor for extracellular ATP-mediated apoptotic cell death [53] and inflammation [54]. *P2RX7* mediates NLRP3 inflammasome activation, cytokine and chemokine release, T lymphocyte survival and differentiation, transcription factor activation, and cell death [55]. In humans, Saunders et al. [48] found that a SNP in the *P2RX7* gene failed to induce apoptosis and abolishes the P2X7-mediated pathway allowing survival of mycobacteria within infected host cells.

The *avidin* (*AVD*), another interesting gene involved in the defense response to bacterium BP, was one the most upregulated in the femur of affected broilers, suggesting a potential role in the resilience of chickens. The *AVD* is a biotin-binding glycoprotein expressed under inflammation in several chicken tissues [56], acting as a defense protein protecting the development of the chicken embryo against microbial infections [57]. Packialakshmi et al. [58] found elevated levels of *Gallinacin-9* (*GAL9*), another chicken defense peptide, in plasma of FHN-affected 35 day-old commercial broilers compared to normal broilers.

The cytokines are a family of secreted proteins involved in immunoregulatory and inflammatory processes. In our study, *C-C motif chemokine ligand 19* (*CCL19*) was one of the most expressed genes in the affected chickens (Table 1). The upregulation of *CCL19* and *CCL21* and their receptor *CCR7* was associated to Rheumatoid arthritis pathogenesis, playing an important role in bone destruction by increasing osteoclast migration and resorption activity [59]. Therefore, genes involved in inflammation could lead to a disruption in the bone development of fast-growing chickens and be another possible cause of FHS.

The increased levels of antimicrobial peptide genes in the growth plate of FHS-affected broilers found in our study could indicate microbial infection of the femur. Femur epiphysiolysis has been implicated in subsequent problems, such as bacterial infection [6]. The femoral head damage compromises the physiological barrier against infections and can lead to FHN and osteomyelitis [15], conditions potentially related to BCO. The BCO could be initiated by mechanical microfracturing of the growth plate, followed by colonization of osteochondrotic clefts by a wide range of different opportunistic bacteria hematogenously dispersed [60]. The most frequent organism associated to osteomyelitis is *Staphylococcus aureus* [61, 62]. The ability of *S. aureus* to infect the osteoblast is highly correlated to its ability to bind to the extracellular matrix, and multiple adhesins produced by the bacteria have strong interaction with components of the avian extracellular matrix [63–65]. After osteoblasts infection, bacterium modulates the production of cytokines and chemokines, together with the increase of osteoclastogenesis and death of osteoblasts, increasing the imbalance of bone homeostasis [64, 65]. Therefore, besides its structural function, the ECM also modulates the microorganism colonization in the growth plate tissue. The immune response BP grouped genes possibly involved with the cause and consequence of the FHS. Those related to defense response to other organism and response to bacteria seem to be a consequence of FHS, while genes enriching inflammatory BP could be triggering this disorder in commercial broilers.

### Apoptosis or cell death

Cell death is a physiological mechanism probably involved in the FHS in broilers due to its essential role in development and tissue homeostasis. The forms of cell death (necrosis, apoptosis and autophagy) are not separated by strict boundaries sharing molecular effectors and signaling ways [66]. In our study, biological process associated to cell death, such as regulation of cell death, regulation of apoptotic process, programmed cell death and regulation of programmed cell death (Additional file 3) grouped *ADA, AGT, CEBPB, G0S2, IRF1, JAK2, OSGIN1, P2RX7* and *PDK4* genes that were upregulated in FHS-affected broilers, while *ADIPOQ, AIFM2, CRIP1, CTSD, GLI2, HSPB1, SFRP1* and *THBS1* were downregulated.

The *P2RX7* gene can be highlighted because its activation stimulates downstream events, triggering the caspase cascade and leading to apoptotic death of the target cell [67]. Another interesting gene related to apoptosis is the *TP53 apoptosis effector* (*PERP2*), which was not recognized in BP with DAVID database. According to KEGG p53 signaling pathway, the p53 activation is induced by a number of stress signals, including DNA damage, oxidative stress and activated oncogenes. The p53 protein is employed as a transcriptional activator of p53-regulated genes resulting in cell cycle arrest, cellular senescence or apoptosis. In our study, *PERP2* was one of the most downregulated genes in the GP transcriptome, being 3.04 times less expressed in the affected than in the normal group. This expression profile was confirmed by the qPCR analysis, with *PERP2* being 16.1 times less expressed in FHS-affected than in normal broilers (Table 2). In chickens, the function of *PERP2* has not been widely studied. However, this gene has already been associated with epithelial diseases in humans, mainly because of its function on epithelial integrity and cell-cell adhesion, due to an impaired intercellular adhesion observed after mechanical stress [68]. This finding is interesting since one of the possible causes of FHS and FHN in broilers is due to mechanical stress triggered by the rapid growth and heavy weight of modern broilers.

The *G0S2* gene was upregulated and was enriched in the apoptosis and cell death pathways (Additional file 3). *G0S2* promotes apoptosis by interacting with the anti-apoptotic protein Bcl-2, and downregulation of *G0S2* expression could be a prime avenue for growing cells, especially cancer cells, in preventing death or growth arrest. Despite the *G0S2* pro-apoptotic function, this mechanism may be cell type-specific. The presence of high levels of *G0S2* does not seem to induce apoptosis in adipocytes, which are known to be quite resilient to apoptotic death [69].

Even though FHS is commonly observed in meat-type chicken, in this case and control study, response to stress, cell death and immune response processes were BP highly represented. The high number of DE genes in these processes gives insight on the subclinical implications of epiphysiolysis in broilers. The disruption of these biological processes may impact femur head development and lead to more severe femur disorders, such as necrosis and osteomyelitis.

This study identified several pathways and biological processes related to FHS, corroborating with the major hypotheses regarding the causes of this disorder. Furthermore, we highlighted that the impaired cell adhesion and ECM seem to be the most important processes to trigger FHS in chickens. These mechanisms have not been widely explored if compared to those related to angiogenesis and bone development. Moreover, DE genes related to inflammation and lipidemia were also enriched in the BP found in our study. The role of these mechanisms in metabolic disorders, including those related to bone, has recently been discussed, especially in humans. It is known that inflammatory diseases are associated to bone losses, bone resorption and obesity [70, 71]. Therefore, the fast growth of broilers, mainly around 35 days of age, could increase the expression of inflammatory and lipid related genes, influencing the development of FHS in broilers. These findings are in agreement with studies that did not find any bone quality issue in fast-growing chickens evaluated in early ages of development.

Understanding the changes in the GP transcriptome might support breeding strategies to obtain methods to address poultry robustness and more resilient broilers. Furthermore, identifying young broilers with vulnerable femoral joint can help genetic selection against FHS. Currently, clinical signs of FHN are not detectable at early stages. The diagnoses of these anomalies are done at late stage due to lameness or after necropsy. Noninvasive biomarkers that can help genetic selection of broilers against FHS are required to assess the predisposition to FHN [13]. Thus, some of the proteins coded by DE genes such as *LOXL2*, *ADA*, *PERP2, ADIPOQ*, *MAPK12, FN1, P2RX7* and *AVD* found in our study should be investigated as biomarkers, which could be easily evaluated in the blood. Moreover, further functional analyses of these genes are interesting to elucidate their contribution to the development of the femur head necrosis in chickens, which would also be important for the understanding of humans bone disorders.

## Conclusions

Genes with potential role in epiphysiolysis were identified through the femur head transcriptome analysis, providing a better understanding of the mechanisms that regulate bone development in fast-growing chickens. In this study, we highlighted the importance of cell adhesion and extracellular matrix related genes in triggering FHS. The imbalance in expression of the genes involved in these processes could make broilers more susceptible to this condition. Furthermore, we have shown that, besides the angiogenesis and bone development genes expected to be DE in FHS, new findings were described associating lipidemia and immune response/inflammation with FHS in broilers.

## Methods

### Experimental animals and sample collection

This study was performed with the approval of the Embrapa Swine and Poultry Ethical Committee of Animal Use (CEUA) under protocol number 012/2012. Commercial male broilers (Cobb500) with 35 days of age from a poultry farm located in Concórdia, Santa Catarina State, Brazil, were used. The chickens were housed according to optimal industry growing standard practices for this line and raised with free access to both feed and water. To reduce environmental effects, broilers used in this study were sampled from the same flock, in a Darkhouse system managed by a high standard producer. Based on visual observation, a total of 29 individuals, including 14 healthy and 15 lameness-affected chickens, were sampled and transferred to the Embrapa Swine and Poultry National Research Center, located in Concordia-SC, for tissue sample collection. The body score was evaluated in all animals prior to necropsy. Broilers were euthanized by cervical dislocation followed by bleeding.

The proximal femoral head was classified according to the presence or absence of different levels of BCO, based upon clinical examination of the growth plate (GP) separation from the articular cartilage (AC) by visual observation of compatible necrosis lesions, according to Wideman et al. [12]. Femoral samples with good adhesion between GP and AC were considered in the normal group. On the other hand, samples presenting separation between GP and AC showing no lesions in GP comprised the proximal femoral head separation (FHS) or epiphysiolysis group. In the proximal femoral head region from all broilers, samples were collected from GP, stored in liquid nitrogen and transferred to the − 80 °C freezer.

### RNA isolation, cDNA library and sequencing

Total RNA was isolated from 100 mg of the femoral head tissue using Trizol Reagent (Invitrogen, Carlsbad, CA) following the manufacturer’s instructions. The RNA cleanup was performed using RNeasy mini kit (Qiagen, Germany) also following the manufacturer’s instructions. Total RNA was quantified in Nanodrop spectrophotometer (Thermo Scientific; Waltham, MA, USA) and the integrity was assessed using an Agilent 2100 BioAnalyzer (Agilent Technologies; Santa Clara, CA, USA). Samples with RNA integrity number (RIN) higher than 8 were considered for further analysis. A total of eight samples of GP, four normal and four FHS-affected were prepared for RNA-sequencing. Barcoded Illumina sequencing libraries for gene expression profile were obtained for each of the 8 individuals using the TruSeq™ RNA Sample Prep Kit v2 (Illumina, Inc.; San Diego CA, USA), according to the manufacturer’s recommendations, with 2 μg of total RNA. Libraries were submitted to the Functional Genomics Center, ESALQ, University of São Paulo, Piracicaba, São Paulo State, Brazil for sequencing in Illumina HiSeq2500 equipment (Illumina, Inc.; San Diego CA, EUA), all in the same lane, following the 2x100bp paired-end protocol.

### Transcriptome analyses

Raw FASTQ reads were submitted to quality control using the SeqyClean tool [72] for removing short reads (< 70 bp), low quality reads (QPhred < 24) and adapter sequences. Sequence reads were mapped to the chicken reference genome (*Gallus gallus*, assembly 5.0) available in Ensembl 87 (www.ensembl.org) using the BWA-MEM software [73]. The counts of fragments were obtained using the HTseq software [74]. The differentially expressed (DE) genes were identified using the edgeR package [75]. The DE genes between FHS-affected and healthy broilers were selected based on the level of False Discovery Rate (FDR ≤ 0.05) after the Benjamini-Hochberg (BH) method for multiple correction tests [76]. To verify the expression pattern between FHS-affected and unaffected groups, the LogFC values from each gene were used to create multidimensional scaling (*MDS*) plots using R. Based on DE genes, a heatmap was generated to check the consistence between samples using R. The FASTQ files obtained in this study were deposited in the SRA database, with BioProject number PRJNA352962.

### Functional analyses

The functional annotation analyses were performed based on the DE genes with FDR < 0.05. The FASTA files of these genes obtained in the Ensembl 87 were submitted to the Blast2GO software [77] for the gene ontology (GO) search. Furthermore, the DAVID database (http://david.abcc.ncifcrf.gov/tools.jsp) was used to improve the biological processes (BP) obtained in the Blast2GO software, where the expressed genes in this study were set as background. Once obtained the BP, the GO numbers from DAVID were clustered by the REViGO tool [78] for easily visualization and understanding. Genes with positive LogFC values were considered upregulated while those with negative values were downregulated in the FHS-affected broilers compared to the normal group. Gene networks were constructed using Stringdb and the NetworkAnalyst tools [79, 80].

### Validation by quantitative PCR (qPCR)

Quantitative PCR analysis was used to confirm the expression pattern of six DE genes in the GP transcriptome using 20 samples of 35 day-old broilers (10 normal and 10 affected). The genes selected were: Low density *lipoprotein receptor-related protein 1B* (*LRP1B*), *Collagen type XXVIII alpha 1 chain* (*Col28A1*), *Family with sequence similarity 180, member A* (*FAM180A*), *TP53 apoptosis effector* (*PERP2*), *Carbohydrate sulfotransferase 1* (*CHST1*) and *Perilipin-1* (*PLIN1*). Those genes were selected based on their LogFC, FDR and function. Sequences were obtained from *Gallus gallus* on Genebank (http://www.ncbi.nlm.nih.gov/gene/) and Ensembl (www.ensembl.org). Primers used for each gene (Table 3) were designed in exon-exon junctions using the Primer-Blast online tool [81]. The primers’ quality was evaluated in the NetPrimer online software (http://www.premierbiosoft.com/netprimer/). The *Ribosomal protein 4* (*RPL4*) and *Ribosomal protein 30* (*RPL30*) genes were used as reference [32]. The qPCR reactions were carried out in QuantStudio 6 (Applied Biosystems, USA) equipment, in a final volume of 15 μL containing 1X Maxima Mastermix SYBR Green (Fermentas, USA), 0.133 μM for each primer and 2 μL of cDNA. The cycle threshold (Ct) mean for each replicate sample was obtained and normalized with the reference genes. The relative quantification analysis was performed using the Relative Expression Software Tool (REST, [82]) that applies a pair wise fixed reallocation randomization test, a nonparametric statistical test.

**Table 1:** Top 10 down and upregulated genes in the proximal femur transcriptome of FHS-affected compared to healthy broilers, according to the LogFC and FDR

Ensembl ID	LogFC	FDR	Gene
ENSGALG00000027184	−5.38	5.00E-10	*OLFML1*
ENSGALG00000032856	−4.92	1.07E-06	*GFRA2*
ENSGALG00000039376	−4.88	7.40E-07	*DPT*
ENSGALG00000024428	−4.84	2.39E-06	*C17orf67*
ENSGALG00000028459	−4.46	1.18E-07	*FAM180A*
ENSGALG00000015307	−4.40	2.19E-07	*ABI3BP*
ENSGALG00000017191	−3.81	2.35E-04	*ANGPTL5*
ENSGALG00000043703	−3.47	1.26E-07	
ENSGALG00000032220	−3.27	8.77E-07	*ELN*^*a*^
ENSGALG00000016511	−3.14	7.71E-03	*ADGRG2*
ENSGALG00000009479	2.20	8.76E-03	*SAMD9L*
ENSGALG00000044811	2.21	3.60E-05	*MSMB-like*^*a*^
ENSGALG00000046192	2.21	5.45E-04	*CCL19*^*a*^
ENSGALG00000026727	2.25	0.0295	*SLC30A10*
ENSGALG00000043860	2.42	0.0030	*PGR2*^*a*^
ENSGALG00000023933	2.42	0.024	*G0S2*
ENSGALG00000043254	2.56	1.11E-03	*EPX*^*a*^
ENSGALG00000028256	2.67	3.58E-03	*CCL19*
ENSGALG00000023622	3.02	7.06E-03	*AVD*
ENSGALG00000032614	3.37	1.51E-04	*CHAT2*

*OLFML1* Olfactomedin like 1, *GFRA2* GDNF family receptor alpha 2, *DPT Dermatopontin*, *C17orf67 Chromosome 17 open reading frame 67 orf67*, *FAM180A* Family with sequence similarity 180 member A, *ABI3BP* ABI family member 3 binding protein, *ANGPTL5* Angiopoietin like 5, *ADGRG2* Adhesion G protein-coupled receptor G2, *SAMD9L* Sterile alpha motif domain containing 9-like, *SLC30A10* Solute carrier family 30 member, *G0S2G0/G1* switch 2*, CCL19:CC:motif chemokine ligand 19*, *AVD* Avidin, *CHAT2* ADP-ribosyltransferase. ^*a*^*Genes annotated in the Ensembl 98 release. ELN: Elastin, MSMB-like: Microseminoprotein Beta Like, CCL19* C-C Motif Chemokine Ligand 19*, PGR2* Progesterone receptor 2*, EPX* Eosinophil Peroxidase

**Table 2:** Relative gene expression between healthy and FHS proximal femur in RNA-Seq and qPCR analyses in 35-day-old broilers

	RNA-Seq		qPCR		
Gene	FDR	LogFC	LogFC	Std. error	*P*-value
*LRP1B*	3.32E-05	−1.98	−13.51	0.01–0.88	0.012
*Col28A1*	6.24E-08	−2.59	−3.62	0.07–1.26	0.013
*PERP2*	1.49E-13	−3.04	−16.10	0.01–0.37	0.001
*FAM180A*	1.18E-07	−4.46	−2.85	0.08–1.41	0.035
*CHST1*	1.81E-05	−2.13	−3.70	0.04–1.55	0.046
*PLIN1*	0.03	2.08	1.82	0.20–18.32	0.427

*LRP1B* Low density lipoprotein receptor-related protein 1B, *Col28A1* Collagen type XXVIII alpha 1 chain, *FAM180A* Family with sequence similarity 180, member A, *PERP2* TP53 apoptosis effector, *CHST1* Carbohydrate sulfotransferase 1 and *PLIN1* Perilipin-1, FDR (False Discovery Rate), LogFC (Log Fold Change)

**Table 3:** Primers of the target and normalizing genes used in the quantitative PCR analyses

Gene	Primers sequence (5′ – 3′)	Ensembl ID
*LRP1B*	F: GGCTACTGCTACAATGGTGGT	ENSGALG00000012407
	R: GTTGGCGGAGCATAGACAGA	
*COL28A1*	F: ACCAGGTCTAAAGGGTCAACG	ENSGALG00000010259
	R: TGGATTCCCAGAGTCTCCCA	
*PERP2*	F: TGGACTATGGATGGGGGAGAG	ENSGALG00000027207
	R: GAGGGCGAAACAGATGACCA	
*FAM180A*	F:GAGTAGAGCTATGCTTTACCCAGC	ENSGALG00000028459
	R: CGAAGCCAGCTCCTCATCTT	
*PLIN1*	F: GGCTATGGAGACGGTGGATG	ENSGALG00000023395
	R: CTGGCTTGCTCTCCTCTTCC	
*CHST1*	F: CGCCCCTCTTTCTCGTCTTC	ENSGALG00000008440
	R: GCTTGGAGAGACCCGATTCC	
RPL4*	F: TGTTTGCCCCAACCAAGACT	ENSGALG00000007711
	R: CTCCTCAATGCGGTGACCTT	
*RPL30*^*a*^	F: ATGATTCGGCAAGGCAAAGC	ENSGALG00000008212
	R: GTCAGAGTCACCTGGGTCAA	

^*a*^Petry et al. [32]

## Supplementary information

**Additional file 1.** Multi-Dimensional Scaling Plot (MDS-plot) measuring the similarity of the samples into 2-dimensions (blue represents the normal group and red the affected group).

**Additional file 2.** An excel file with the expressed and DE genes between normal and FHS-affected broilers with 35 days of age.

**Additional file 3.** GO biological process enriched based on the differentially expressed genes between normal and FHS-affected broilers.

## Data Availability

The datasets used or analyzed during the current study are available from the corresponding author on reasonable request. The transcriptome sequences are available in the SRA database with BioProject number PRJNA352962 and biosample numbers: SAMN06007242, SAMN06007241, SAMN06007240, SAMN06007239, SAMN06007238, SAMN06007237, SAMN06007236 and SAMN06007235.

## Abbreviations

AC: Articular cartilage
ATP: Adenosine triphosphate
BCO: Bacterial Chondronecrosis with Osteomyelitis
BP: Biological processes
cAMP: Adenosine 3′,5′monophosphate
CEUA: Ethical Committee of Animal Use
Ct: Cycle threshold
DE: Differentially expressed
ECM: Extracellular matrix
FDR: False Discovery Rate
FGFs: Fibroblast growth factors
FHN: Femoral Head Necrosis
FHS: Femoral head separation
GAGs: Glycosaminoglycans
GP: Growth plate
LogFC: Log fold change
MDS: Multi-dimensional scaling
MF: Molecular functions
qPCR: Quantitative PCR
