# Supplementary figures and images for: Proximal femoral head transcriptome reveals novel candidate genes related to epiphysiolysis in broiler chickens
Source: BMC Genomics. 2019 Dec 30;20:1031. doi: 10.1186/s12864-019-6411-9 (PMC6937697; doi:10.1186/s12864-019-6411-9)

# MDS

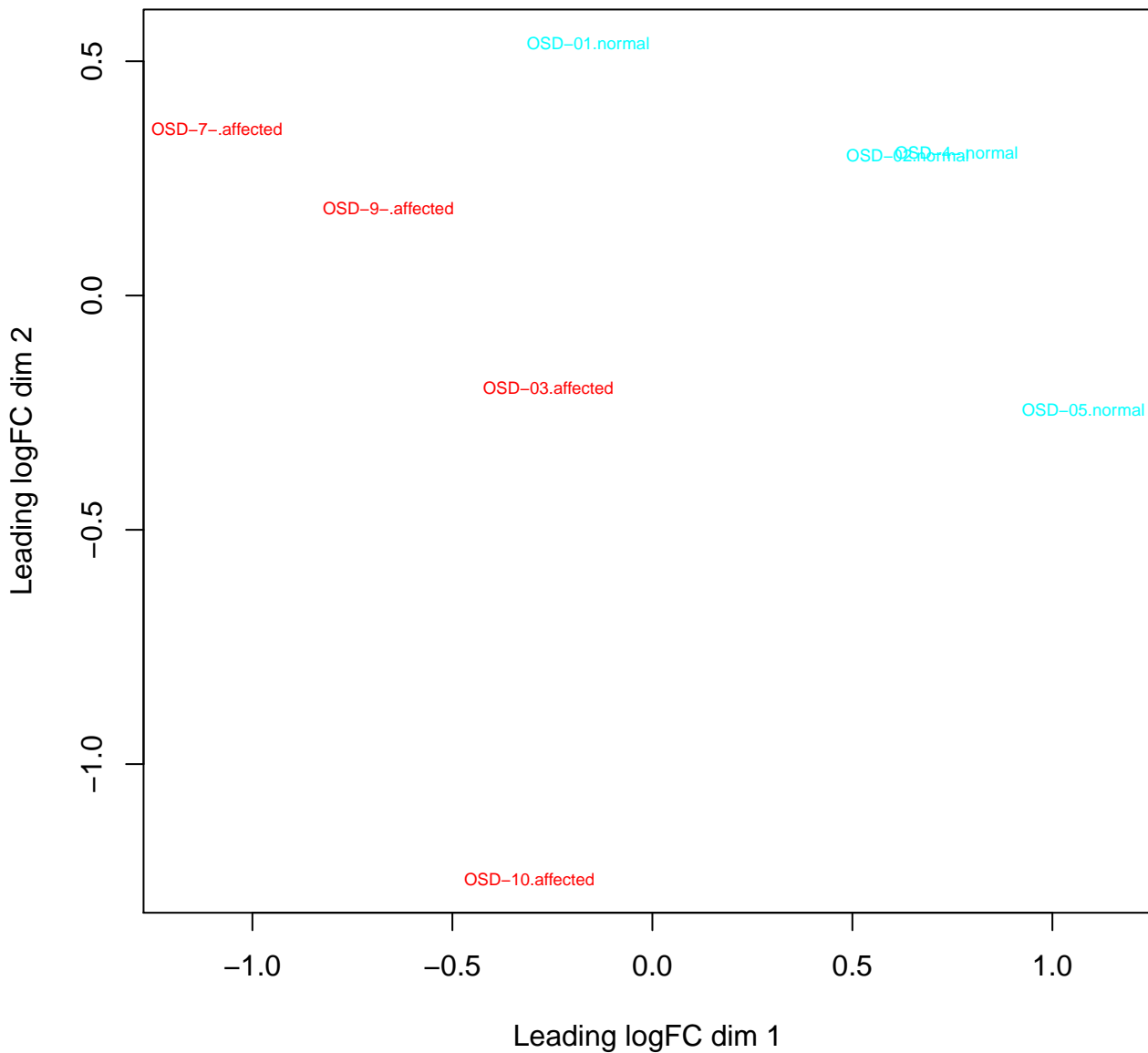

Supplement: Supplementary file 1 — Additional file 1. Multi-Dimensional Scaling Plot (MDS-plot) measuring the similarity of the samples into 2-dimensions (blue represents the normal group and red the affected group). [file 12864_2019_6411_MOESM1_ESM.pdf]
