# Supplementary material for: Proximal femoral head transcriptome reveals novel candidate genes related to epiphysiolysis in broiler chickens
Source: BMC Genomics. 2019 Dec 30;20:1031. doi: 10.1186/s12864-019-6411-9 (PMC6937697; doi:10.1186/s12864-019-6411-9)
Supplement: Supplementary file 3 — Additional file 3. GO biological process enriched based on the differentially expressed genes between normal and FHS-affected broilers. [file 12864_2019_6411_MOESM3_ESM.docx]

**Additional File 3. Bioprocesses of differentially expressed genes between FHS-affected and unaffected broilers.**

| **DAVID Bioprocesses** | **Enriched genes** |
| --- | --- |
| GO:0000902 cell morphogenesis | *FRYL, EPB42, KEL, TNMD, LPAR1, IL7R, GLI2, OGN, P2RX7, APOA1, SFRP1, TENM2, TBC1D7, LOXL2, FN1, KIF26B* |
| GO:0000904 cell morphogenesis involved in differentiation | *OGN, APOA1, SFRP1, TENM2, KEL, TNMD, LOXL2, GLI2, KIF26B, FN1* |
| GO:0001101 response to acid chemical | *CEBPB, PDK4, PDGFRA, COL6A1, COL16A1, CBS* |
| GO:0001501 skeletal system development | *P2RX7, SFRP1, PDGFRA, CYTL1, PRRX1, NPR3, LOXL2, GLI2, CBS* |
| GO:0001503 ossification | *P2RX7, CEBPB, SFRP1, ITGA11, COL6A1, PTCH1, GLI2, SLC26A2, CBS* |
| GO:0001525 angiogenesis | *MEOX2, TNMD, PDGFRA, HSPB1, LOXL2, COL8A1, THBS1* |
| GO:0001649 osteoblast differentiation | *CEBPB, SFRP1, ITGA11, COL6A1, PTCH1, GLI2* |
| GO:0001655 urogenital system development | *SFRP1, AGT, PDGFRA, PTCH1, GLI2, ADIPOQ, KIF26B* |
| GO:0001709 cell fate determination | *PRRX1, PTCH1* |
| GO:0001763 morphogenesis of a branching structure | *SFRP1, AGT, PTCH1, GLI2* |
| GO:0001775 cell activation | *P2RX7, CHGA, CEBPB, SFRP1, IRF1, HSPB1, RSAD2, IL7R, THBS1, CD151, ADA, MYL9* |
| GO:0001816 cytokine production | *P2RX7, CHGA, APOA1, CEBPB, IRF1, HSPB1, RSAD2, THBS1, ADIPOQ, FN1* |
| GO:0001935 endothelial cell proliferation | *APOA1, TNMD, LOXL2, THBS1* |
| GO:0002252 immune effector process | *NLRC5, P2RX7, CHGA, APOA1, CEBPB, PDK4, IRF1, RSAD2, IL7R, ADA* |
| GO:0001822 kidney development | *SFRP1, AGT, PDGFRA, PTCH1, GLI2, ADIPOQ, KIF26B* |
| GO:0002252 immune effector process | *NLRC5, P2RX7, CHGA, APOA1, CEBPB, PDK4, IRF1, RSAD2, IL7R, ADA* |
| GO:0002376 immune system process | *C7, RSAD2, CD151, IL7R, ADA, NLRC5, GFI1B, APOA1, THBS1, FYB, PTPRC, CEBPB, EPB42, PDK4, TINAGL1, ADIPOQ, AMPD3, RHAG, P2RX7, CHGA, SFRP1, AVD, PDGFRA, IRF1, JAK2, MFAP5* |
| GO:0002456 T cell mediated immunity | *P2RX7, RSAD2, IL7R* |
| GO:0002520 immune system development | *GFI1B, CEBPB, SFRP1, EPB42, IRF1, PDGFRA, RSAD2, JAK2, IL7R, RHAG, MFAP5, ADIPOQ, ADA* |
| GO:0002521 leukocyte differentiation | *CEBPB, SFRP1, IRF1, RSAD2, IL7R, ADIPOQ, ADA* |
| GO:0002544 chronic inflammatory response | *CEBPB, THBS1* |
| GO:0002682 regulation of immune system process | *NLRC5, PTPRC, P2RX7, CEBPB, APOA1, SFRP1, PDK4, IRF1, RSAD2, THBS1, IL7R, ADIPOQ, ADA* |
| GO:0002683 negative regulation of immune system process | *NLRC5, APOA1, CEBPB, SFRP1, IRF1, IL7R, THBS1, ADIPOQ, ADA* |
| GO:0003014 renal system process | *AGT, NPR3, ADIPOQ* |
| GO:0006022 aminoglycan metabolic process | *XYLT1, CYTL1, ITIH5, CHST1* |
| GO:0006154 adenosine catabolic process | *CECR1, ADA* |
| GO:0006638 neutral lipid metabolic process | *SREBF1, APOA1, PNPLA2* |
| GO:0006641 triglyceride metabolic process | *SREBF1, APOA1, PNPLA2* |
| GO:0006783 heme biosynthetic process | *CPOX, UROS* |
| GO:0006869 lipid transport | *ABCA8, P2RX7, APOA1, OSBPL1A, PTCH1, THBS1, ADIPOQ* |
| GO:0006915 apoptotic process | *CRIP1, CEBPB, AIFM2, PDK4, OSGIN1, GLI2, ADIPOQ, ADA, P2RX7, SFRP1, AGT, IRF1, HSPB1, G0S2, THBS1* |
| GO:0006928 movement of cell or subcellular component | *MLPH, ITGA11, RASGEF1A, LPAR1, CD151, GLI2, ADIPOQ, ADA, OGN, CHGA, APOA1, SFRP1, AGT, TENM2, PDGFRA, HSPB1, JAK2, THBS1, LOXL2, FN1, KIF26B* |
| GO:0006935 chemotaxis | *OGN, CHGA, TENM2, PDGFRA, HSPB1, LPAR1, GLI2, THBS1, KIF26B* |
| GO:0006952 defense response | *CEBPB, PDK4, RSAD2, ADIPOQ, ADA, NLRC5, P2RX7, CHGA, APOA1, ALOX5AP, AVD, IRF1, JAK2, THBS1, FN1* |
| GO:0006954 inflammatory response | *P2RX7, APOA1, CEBPB, ALOX5AP, JAK2, THBS1, ADIPOQ, ADA, FN1* |
| GO:0006955 immune response | *FYB, C7, PTPRC, RSAD2, IL7R, TINAGL1, ADA, NLRC5, P2RX7, CHGA, APOA1, AVD, IRF1, JAK2, THBS1* |
| GO:0007155 cell adhesion | *CEBPB, ITGA11, RSAD2, IL7R, COL16A1, CD151, VIT, TINAGL1, ADIPOQ, ADA, ABI3BP, MYL9, P2RX7, APOA1, CD34, TENM2, COL6A3, IRF1, COL6A1, HSPB1, JAK2, THBS1, COL8A1, KIF26B, FN1* |
| GO:0007160 cell-matrix adhesion | *CD34, ITGA11, THBS1, COL16A1, FN1* |
| GO:0007166 cell surface receptor signaling pathway | *CREBRF, PRRX1, ITGA11, CD151, GLI2, ADA, NLRC5, OGN, APOA1, AGT, THBS1, RHBDF2, SREBF1, PTPRC, TRABD2B, PDK4, ADIPOQ, RASL11B, P2RX7, SFRP1, PDGFRA, IRF1, HSPB1, PTCH1, JAK2, G0S2* |
| GO:0007167 enzyme linked receptor protein signaling pathway | *RASL11B, RHBDF2, SREBF1, SFRP1, AGT, PDK4, PDGFRA, HSPB1, JAK2, THBS1, ADIPOQ* |
| GO:0007169 transmembrane receptor protein tyrosine kinase signaling pathway | *RHBDF2, SREBF1, AGT, PDK4, PDGFRA, HSPB1, JAK2, THBS1, ADIPOQ* |
| GO:0007186 G-protein coupled receptor signaling pathway | *ADCY2, APOA1, SFRP1, AGT, NPR3, LPAR1, ADA* |
| GO:0007193 adenylate cyclase-inhibiting G-protein coupled receptor signaling pathway | *ADCY2, NPR3, LPAR1* |
| GO:0007224 smoothened signaling pathway | *SFRP1, PRRX1, PTCH1, GLI2* |
| GO:0007369 gastrulation | *APOA1, SFRP1, COL6A1, COL8A1, ADIPOQ, FN1* |
| GO:0007492 endoderm development | *COL6A1, COL8A1, FN1* |
| GO:0008015 blood circulation | *SREBF1, AGT, NPR3, ADIPOQ, ADA, CBS* |
| GO:0008219 cell death | *CRIP1, CEBPB, AIFM2, PDK4, OSGIN1, GLI2, ADIPOQ, ADA, P2RX7, MEOX2, SFRP1, AGT, IRF1, CTSD, HSPB1, JAK2, G0S2, THBS1* |
| GO:0008283 cell proliferation | *CEBPB, TNMD, PRRX1, NPR3, CD151, IL7R, GLI2, ADIPOQ, ADA, P2RX7, APOA1, SFRP1, IRF1, PTCH1, THBS1, COL8A1, LOXL2, ACSL6, FN1* |
| GO:0008589 regulation of smoothened signaling pathway | *SFRP1, PRRX1, PTCH1, GLI2* |
| GO:0009112 nucleobase metabolic process | *ALDH6A1, CECR1, ADA* |
| GO:0009306 protein secretion | *RHBDF2, SREBF1, P2RX7, APOA1, SFRP1, RSAD2, FN1* |
| GO:0009607 response to biotic stimulus | *NLRC5, P2RX7, CHGA, CEBPB, AVD, PDK4, IRF1, HSPB1, RSAD2* |
| GO:0009719 response to endogenous stimulus | *RASL11B, SREBF1, CREBRF, P2RX7, CEBPB, SFRP1, PDK4, PDGFRA, COL6A1, JAK2, THBS1, COL16A1, ADIPOQ, CBS* |
| GO:0009790 embryo development | *MYH15, PRRX1, LRIG3, GLI2, ADIPOQ, ADA, APOA1, MEOX2, SFRP1, PDGFRA, COL6A1, PTCH1, COL8A1, FN1* |
| GO:0009792 embryo development ending in birth or egg hatching | *MYH15, MEOX2, SFRP1, PDGFRA, PRRX1, PTCH1, GLI2, FN1* |
| GO:0009887 organ morphogenesis | *P2RX7, CEBPB, SFRP1, AGT, PDGFRA, PRRX1, PTCH1, LRIG3, GLI2, COL8A1, CBS, KIF26B, FN1* |
| GO:0009888 tissue development | *MYH15, KEL, TNMD, CYTL1, PRRX1, GLI2, ADIPOQ, ADA, FAM65B, P2RX7, MEOX2, TAGLN, SFRP1, AGT, BNC2, PDGFRA, COL6A1, PTCH1, COL8A1, LOXL2, CBS, FN1, KIF26B* |
| GO:0009966 regulation of signal transduction | *RHBDF2, CREBRF, TRABD2B, PRRX1, RSAD2, RASGEF1A, LPAR1, GLI2, ADIPOQ, ADA, RASL11B, NLRC5, P2RX7, APOA1, SFRP1, AGT, IRF1, HSPB1, TBC1D7, JAK2, G0S2, PTCH1, THBS1, CBS, FN1* |
| GO:0009967 positive regulation of signal transduction | *CREBRF, NLRC5, P2RX7, APOA1, SFRP1, AGT, PRRX1, RSAD2, RASGEF1A, JAK2, G0S2, LPAR1, THBS1, ADIPOQ, ADA* |
| GO:0009968 negative regulation of signal transduction | *RASL11B, CREBRF, NLRC5, P2RX7, APOA1, SFRP1, AGT, TRABD2B, HSPB1, TBC1D7, PTCH1, THBS1, ADIPOQ, ADA* |
| GO:0009991 response to extracellular stimulus | *SREBF1, P2RX7, SFRP1, PDK4, CBS* |
| GO:0010033 response to organic substance | *CREBRF, SREBF1, CEBPB, PDK4, COL16A1, GLI2, ADIPOQ, RASL11B, NLRC5, P2RX7, APOA1, SFRP1, AGT, IRF1, PDGFRA, COL6A1, HSPB1, TBC1D7, JAK2, PTCH1, THBS1, CBS* |
| GO:0010035 response to inorganic substance | *P2RX7, CRIP1, PTCH1, LOXL2, THBS1, ITPR3* |
| GO:0010038 response to metal ion | *P2RX7, CRIP1, LOXL2, THBS1, ITPR3* |
| GO:0010243 response to organonitrogen compound | *SREBF1, P2RX7, CEBPB, SFRP1, PDK4, PDGFRA, COL6A1, JAK2, COL16A1, ADIPOQ, CBS* |
| GO:0010565 regulation of cellular ketone metabolic process | *SREBF1, APOA1, PDK4, ADIPOQ* |
| GO:0010631 epithelial cell migration | *APOA1, AGT, HSPB1, LOXL2, THBS1* |
| GO:0010646 regulation of cell communication | *CREBRF, SLC6A2, PRRX1, RASGEF1A, RSAD2, LPAR1, GLI2, ADA, NLRC5, APOA1, AGT, FN1,TBC1D7, THBS1, SREBF1, RHBDF2, CBS, TRABD2B, ITPR3, ADIPOQ, RASL11B, P2RX7, SFRP1, IRF1, HSPB1, JAK2, PTCH1, G0S2* |
| GO:0010647 positive regulation of cell communication | *CREBRF, SLC6A2, PRRX1, RASGEF1A, RSAD2, LPAR1, ITPR3, ADIPOQ, ADA, NLRC5, P2RX7, APOA1, SFRP1, AGT, JAK2, G0S2, THBS1* |
| GO:0010648 negative regulation of cell communication | *CREBRF, SREBF1, TRABD2B, ADIPOQ, ADA, RASL11B, NLRC5, P2RX7, APOA1, SFRP1, AGT, HSPB1, TBC1D7, PTCH1, THBS1* |
| GO:0010876 lipid localization | *ABCA8, P2RX7, APOA1, OSBPL1A, PNPLA2, PTCH1, THBS1, ADIPOQ* |
| GO:0010941 regulation of cell death | *P2RX7, CEBPB, AIFM2, SFRP1, AGT, PDK4, HSPB1, OSGIN1, JAK2, G0S2, GLI2, THBS1, ADIPOQ, ADA* |
| GO:0010942 positive regulation of cell death | *P2RX7, AIFM2, SFRP1, AGT, OSGIN1, G0S2, THBS1, ADIPOQ* |
| GO:0012501 programmed cell death | *CRIP1, CEBPB, AIFM2, PDK4, OSGIN1, GLI2, ADIPOQ, ADA, P2RX7, SFRP1, AGT, IRF1, CTSD, HSPB1, JAK2, G0S2, THBS1* |
| GO:0015850 organic hydroxy compound transport | *CHGA, APOA1, SLC6A2, PTCH1, ADIPOQ* |
| GO:0016337 single organismal cell-cell adhesion | *P2RX7, APOA1, CEBPB, TENM2, IRF1, HSPB1, RSAD2, IL7R, CD151, ADIPOQ, ADA, MYL9, KIF26B* |
| GO:0016477 cell migration | *ITGA11, RASGEF1A, LPAR1, CD151, ADIPOQ, ADA, CHGA, APOA1, SFRP1, AGT, PDGFRA, HSPB1, JAK2, THBS1, LOXL2, FN1* |
| GO:0018149 peptide cross-linking | *EPB42, THBS1, FN1* |
| GO:0018158 protein oxidation | *APOA1, TRABD2B* |
| GO:0019216 regulation of lipid metabolic process | *SREBF1, APOA1, AGT, PDK4, PNPLA2, ADIPOQ* |
| GO:0019218 regulation of steroid metabolic process | *SREBF1, APOA1, AGT* |
| GO:0019932 second-messenger-mediated signaling | *ADCY2, TENM2, NPR3, THBS1, ADA* |
| GO:0019935 cyclic-nucleotide-mediated signaling | *ADCY2, NPR3, THBS1* |
| GO:0022008 neurogenesis | *OGN, CEBPB, APOA1, SFRP1, FRYL, TENM2, KEL, PRRX1, PTCH1, LPAR1, GLI2, ACSL6, KIF26B, FN1* |
| GO:0022603 regulation of anatomical structure morphogenesis | *OGN, APOA1, SFRP1, AGT, KEL, TNMD, HSPB1, TBC1D7, LPAR1, THBS1, ADIPOQ, ADA, FN1* |
| GO:0022610 biological adhesion | *CEBPB, ITGA11, RSAD2, IL7R, COL16A1, CD151, VIT, TINAGL1, ADIPOQ, ADA, ABI3BP, MYL9, P2RX7, APOA1, CD34, TENM2, COL6A3, IRF1, COL6A1, HSPB1, JAK2, THBS1, COL8A1, KIF26B, FN1* |
| GO:0023051 regulation of signaling | *CREBRF, SLC6A2, PRRX1, RASGEF1A, RSAD2, LPAR1, GLI2, ADA, NLRC5, APOA1, AGT, TBC1D7, THBS1, FN1, SREBF1, RHBDF2, CBS, TRABD2B, ITPR3, ADIPOQ, RASL11B, P2RX7, SFRP1, IRF1, HSPB1, JAK2, PTCH1, G0S2* |
| GO:0023056 positive regulation of signaling | *CREBRF, SLC6A2, PRRX1, RASGEF1A, RSAD2, LPAR1, ITPR3, ADIPOQ, ADA, NLRC5, P2RX7, APOA1, SFRP1, AGT, JAK2, G0S2, THBS1* |
| GO:0023057 negative regulation of signaling | *CREBRF, SREBF1, TRABD2B, ADIPOQ, ADA, RASL11B, NLRC5, P2RX7, APOA1, SFRP1, AGT, HSPB1, TBC1D7, PTCH1, THBS1* |
| GO:0030097 hemopoiesis | *GFI1B, CEBPB, SFRP1, PDGFRA, IRF1, RSAD2, JAK2, IL7R, MFAP5, ADIPOQ, RHAG, ADA* |
| GO:0030154 cell differentiation | *MYH15, FRYL, KEL, CYTL1, ITGA11, PRRX1, RSAD2, LPAR1, GLI2, IL7R, ADA, FAM65B, OGN, APOA1, COL6A1, LOXL2, COL8A1, ACSL6, FN1, SREBF1, ALDH6A1, RBM24, CEBPB, TNMD, RHAG, ADIPOQ, TAGLN, SFRP1, MAPK12, TENM2, PDGFRA, IRF1, PTCH1, RBM38, JAK2, TLL1, KIF26B* |
| GO:0030155 regulation of cell adhesion | *CEBPB, VIT, IL7R, COL16A1, ADIPOQ, ADA, ABI3BP, APOA1, IRF1, JAK2, THBS1, COL8A1, FN1, KIF26B* |
| GO:0030182 neuron differentiation | *OGN, CEBPB, APOA1, SFRP1, FN1,FRYL, KEL,TENM2, PRRX1, PTCH1, LPAR1, GLI2, KIF26B,* |
| GO:0030198 extracellular matrix organization | *AGT, PDGFRA, LOXL2, VIT, MFAP5, ABI3BP* |
| GO:0030203 glycosaminoglycan metabolic process | *XYLT1, CYTL1, ITIH5, CHST1* |
| GO:0030301 cholesterol transport | *APOA1, PTCH1, ADIPOQ* |
| GO:0031175 neuron projection development | *OGN, APOA1, FRYL, TENM2, KEL, PRRX1, LPAR1, GLI2, KIF26B, FN1* |
| GO:0031344 regulation of cell projection organization | *OGN, P2RX7, SFRP1, TENM2, KEL, PRRX1, TBC1D7, LPAR1, FN1* |
| GO:0031347 regulation of defense response | *NLRC5, APOA1, PDK4, IRF1, RSAD2, JAK2, ADIPOQ, ADA* |
| GO:0031348 negative regulation of defense response | *NLRC5, APOA1, ADIPOQ, ADA* |
| GO:0031589 cell-substrate adhesion | *APOA1, CD34, ITGA11, JAK2, COL8A1, THBS1, COL16A1, VIT, ABI3BP, FN1* |
| GO:0032147 activation of protein kinase activity | *P2RX7, AGT, JAK2, LPAR1, THBS1* |
| GO:0032261 purine nucleotide salvage | *AMPD3, ADA* |
| GO:0032368 regulation of lipid transport | *P2RX7, APOA1, PTCH1, THBS1, ADIPOQ* |
| GO:0032640 tumor necrosis factor production | *HSPB1, THBS1, ADIPOQ* |
| GO:0032940 secretion by cell | *RHBDF2, SREBF1, P2RX7, CHGA, APOA1, SFRP1, STXBP6, SLC6A2, RSAD2, FN1* |
| GO:0032943 mononuclear cell proliferation | *P2RX7, CEBPB, IRF1, IL7R, CD151, ADA* |
| GO:0032989 cellular component morphogenesis | *FRYL, EPB42, KEL, TNMD, LPAR1, IL7R, GLI2, OGN, P2RX7, APOA1, SFRP1, TENM2, PDGFRA, TBC1D7, LOXL2, FN1, KIF26B* |
| GO:0033627 cell adhesion mediated by integrin | *ITGA11, COL16A1, ADA* |
| GO:0034097 response to cytokine | *CREBRF, NLRC5, APOA1, SFRP1, IRF1, JAK2, ADIPOQ* |
| GO:0034433 steroid esterification | *APOA1, AGT* |
| GO:0034435 cholesterol esterification | *APOA1, AGT* |
| GO:0035239 tube morphogenesis | *SFRP1, AGT, PDGFRA, PTCH1, GLI2, KIF26B* |
| GO:0035790 platelet-derived growth factor receptor-alpha signaling pathway | *PDGFRA, ADIPOQ* |
| GO:0035987 endodermal cell differentiation | *COL6A1, COL8A1, FN1* |
| GO:0040011 locomotion | *ITGA11, RASGEF1A, LPAR1, CD151, GLI2, ADIPOQ, ADA, OGN, CHGA, APOA1, SFRP1, AGT, TENM2, PDGFRA, HSPB1, JAK2, THBS1, LOXL2, FN1, KIF26B* |
| GO:0042107 cytokine metabolic process | *CEBPB, IRF1, HSPB1, THBS1* |
| GO:0042180 cellular ketone metabolic process | *SREBF1, APOA1, PDK4, ADIPOQ* |
| GO:0042330 taxis | *OGN, CHGA, TENM2, PDGFRA, HSPB1, LPAR1, GLI2, THBS1, KIF26B* |
| GO:0042440 pigment metabolic process | *CPOX, UROS, CECR1, ADA* |
| GO:0042493 response to drug | *P2RX7, SFRP1, THBS1, ADIPOQ* |
| GO:0042692 muscle cell differentiation | *FAM65B, RBM24, MYH15, APOA1, MAPK12, KEL, PDGFRA, RBM38* |
| GO:0042742 defense response to bacterium | *P2RX7, CHGA, CEBPB, AVD* |
| GO:0042981 regulation of apoptotic process | *P2RX7, CEBPB, AIFM2, SFRP1, AGT, PDK4, HSPB1, OSGIN1, JAK2, G0S2, GLI2, THBS1, ADIPOQ, ADA* |
| GO:0043009 chordate embryonic development | *MEOX2, SFRP1, PDGFRA, PRRX1, PTCH1, GLI2, FN1* |
| GO:0043062 extracellular structure organization | *AGT, PDGFRA, LOXL2, VIT, MFAP5, ABI3BP* |
| GO:0043065 positive regulation of apoptotic process | *P2RX7, AIFM2, SFRP1, AGT, OSGIN1, G0S2, THBS1, ADIPOQ* |
| GO:0043067 regulation of programmed cell death | *P2RX7, CEBPB, AIFM2, SFRP1, AGT, PDK4, HSPB1, OSGIN1, JAK2, G0S2, GLI2, THBS1, ADIPOQ, ADA* |
| GO:0043094 cellular metabolic compound salvage | *CECR1, AMPD3, ADA* |
| GO:0043101 purine-containing compound salvage | *CECR1, AMPD3, ADA* |
| GO:0043200 response to amino acid | *CEBPB, PDGFRA, COL6A1, COL16A1* |
| GO:0043207 response to external biotic stimulus | *NLRC5, P2RX7, CHGA, CEBPB, AVD, PDK4, IRF1, HSPB1, RSAD2* |
| GO:0043405 regulation of MAP kinase activity | *P2RX7, SFRP1, LPAR1, THBS1, ADIPOQ, CBS* |
| GO:0043549 regulation of kinase activity | *NLRC5, P2RX7, SFRP1, AGT, HSPB1, JAK2, LPAR1, THBS1, ADIPOQ, CBS* |
| GO:0044087 regulation of cellular component biogenesis | *P2RX7, CHGA, APOA1, CEBPB, STXBP6, TENM2, AGT, TRABD2B, HSPB1, TBC1D7, LPAR1, COL16A1* |
| GO:0044089 positive regulation of cellular component biogenesis | *P2RX7, CHGA, APOA1, CEBPB, TENM2, AGT, TRABD2B, LPAR1, COL16A1* |
| GO:0045165 cell fate commitment | *CEBPB, SFRP1, PRRX1, PTCH1, GLI2* |
| GO:0045321 leukocyte activation | *P2RX7, CHGA, CEBPB, SFRP1, IRF1, RSAD2, IL7R, THBS1, CD151, ADA* |
| GO:0045444 fat cell differentiation | *SREBF1, ALDH6A1, CEBPB, SFRP1, ADIPOQ* |
| GO:0045595 regulation of cell differentiation | *RBM24, CEBPB, KEL, PRRX1, LPAR1, IL7R, GLI2, ADIPOQ, ADA, OGN, APOA1, SFRP1, IRF1, RBM38, PTCH1, LOXL2, FN1* |
| GO:0045597 positive regulation of cell differentiation | *APOA1, CEBPB, SFRP1, PTCH1, LOXL2, GLI2, IL7R, ADIPOQ, ADA, FN1* |
| GO:0045785 positive regulation of cell adhesion | *APOA1, JAK2, IL7R, COL8A1, COL16A1, VIT, ADA, ABI3BP, KIF26B, FN1* |
| GO:0045859 regulation of protein kinase activity | *P2RX7, SFRP1, AGT, HSPB1, JAK2, LPAR1, THBS1, ADIPOQ, CBS* |
| GO:0046085 adenosine metabolic process | *CECR1, ADA* |
| GO:0046100 hypoxanthine metabolic process | *CECR1, ADA* |
| GO:0046102 inosine metabolic process | *CECR1, ADA* |
| GO:0046103 inosine biosynthetic process | *CECR1, ADA* |
| GO:0046148 pigment biosynthetic process | *CPOX, UROS, CECR1, ADA* |
| GO:0046579 positive regulation of Ras protein signal transduction | *APOA1, RASGEF1A, LPAR1* |
| GO:0046649 lymphocyte activation | *P2RX7, CEBPB, SFRP1, IRF1, RSAD2, IL7R, CD151, ADA* |
| GO:0046849 bone remodeling | *P2RX7, SFRP1, PDK4* |
| GO:0046903 secretion | *RHBDF2, SREBF1, P2RX7, CHGA, APOA1, SFRP1, STXBP6, SLC6A2, RSAD2, ADA, FN1* |
| GO:0048468 cell development | *MYH15, FRYL, KEL, TNMD, PRRX1, LPAR1, GLI2, RHAG, ADIPOQ, FAM65B, OGN, APOA1, SFRP1, TENM2, PDGFRA, LOXL2, FN1, KIF26B* |
| GO:0048513 animal organ development | *MYH15, CYTL1, PRRX1, RSAD2, LRIG3, IL7R, GLI2, MFAP5, FAM65B, GFI1B, APOA1, AGT, LOXL2, COL8A1, FN1, CEBPB, CBS, KIF26B, KEL,EPB42, ADIPOQ, RHAG, P2RX7, SFRP1, MEOX2, BNC2, IRF1, PDGFRA, PTCH1, JAK2, ADA* |
| GO:0048534 hematopoietic or lymphoid organ development | *GFI1B, CEBPB, SFRP1, EPB42, IRF1, PDGFRA, RSAD2, JAK2, IL7R, RHAG, MFAP5, ADIPOQ, ADA* |
| GO:0048565 digestive tract development | *SFRP1, PDGFRA, GLI2, ADA* |
| GO:0048568 embryonic organ development | *PDGFRA, PRRX1, PTCH1, LRIG3, GLI2, ADA* |
| GO:0048584 positive regulation of response to stimulus | *CREBRF, PTPRC, PRRX1, RSAD2, RASGEF1A, LPAR1, ADIPOQ, ADA, NLRC5, P2RX7, APOA1, SFRP1, AGT, IRF1, HSPB1, JAK2, G0S2, THBS1* |
| GO:0048585 negative regulation of response to stimulus | *CREBRF, TRABD2B, IL7R, ADIPOQ, ADA, RASL11B, NLRC5, P2RX7, APOA1, SFRP1, AGT, HSPB1, TBC1D7, PTCH1, THBS1* |
| GO:0048598 embryonic morphogenesis | *APOA1, SFRP1, PDGFRA, PRRX1, COL6A1, PTCH1, LRIG3, GLI2, COL8A1, ADIPOQ, FN1* |
| GO:0048646 anatomical structure formation involved in morphogenesis | *TNMD, GLI2, ADA, SFRP1, MEOX2, PDGFRA, COL6A1, HSPB1, TBC1D7, PTCH1, THBS1, COL8A1, LOXL2, FN1, KIF26B* |
| GO:0048699 generation of neurons | *OGN, CEBPB, APOA1, SFRP1, FRYL, TENM2, KEL, PRRX1, PTCH1, LPAR1, GLI2, ACSL6, KIF26B, FN1* |
| GO:0048705 skeletal system morphogenesis | *P2RX7, SFRP1, PDGFRA, PRRX1, CBS* |
| GO:0048731 system development | *MYH15, FRYL, KEL, CYTL1, PRRX1, RSAD2, LPAR1, LRIG3, GLI2, IL7R, ADA, FAM65B, OGN, APOA1, GFI1B, AGT, LOXL2, COL8A1, THBS1, ACSL6, FN1, CEBPB, EPB42, TNMD, NPR3, RHAG, ADIPOQ, P2RX7, SFRP1, MEOX2, BNC2, TENM2, PDGFRA, IRF1, HSPB1, PTCH1, JAK2, MFAP5, CBS, KIF26B* |
| GO:0048732 gland development | *APOA1, CEBPB, SFRP1, PDGFRA, GLI2, ADA* |
| GO:0048771 tissue remodeling | *P2RX7, SFRP1, PDK4, CBS* |
| GO:0048870 cell motility | *ITGA11, RASGEF1A, LPAR1, CD151, ADIPOQ, ADA, CHGA, APOA1, SFRP1, AGT, PDGFRA, HSPB1, JAK2, THBS1, LOXL2, FN1* |
| GO:0048871 multicellular organismal homeostasis | *P2RX7, CYTL1, HSPB1, AMPD3, RHAG* |
| GO:0048872 homeostasis of number of cells | *P2RX7, JAK2, IL7R, AMPD3, RHAG, ADA* |
| GO:0048878 chemical homeostasis | *P2RX7, APOA1, EPB42, KEL, PDK4, PNPLA2, PTCH1, LPAR1, ITPR3, SLC26A2, ADIPOQ, RHAG* |
| GO:0050673 epithelial cell proliferation | *APOA1, CEBPB, SFRP1, TNMD, PTCH1, LOXL2, COL8A1, THBS1* |
| GO:0050776 regulation of immune response | *NLRC5, PTPRC, P2RX7, APOA1, IRF1, RSAD2, IL7R, ADA* |
| GO:0050777 negative regulation of immune response | *NLRC5, APOA1, IL7R* |
| GO:0050793 regulation of developmental process | *RBM24, CEBPB, KEL, TNMD, PRRX1, LPAR1, IL7R, GLI2, ADIPOQ, ADA, OGN, P2RX7, APOA1, SFRP1, AGT, IRF1, HSPB1, TBC1D7, RBM38, PTCH1, THBS1, LOXL2, FN1* |
| GO:0050801 ion homeostasis | *P2RX7, EPB42, KEL, PDK4, LPAR1, ITPR3, SLC26A2, RHAG* |
| GO:0050873 brown fat cell differentiation | *ALDH6A1, CEBPB, ADIPOQ* |
| GO:0051046 regulation of secretion | *RHBDF2, SREBF1, P2RX7, CHGA, APOA1, SFRP1, SLC6A2, RSAD2, ADA, FN1* |
| GO:0051049 regulation of transport | *SREBF1, RHBDF2, SLC6A2, KEL, RSAD2, REEP1, ADIPOQ, ADA, P2RX7, CHGA, APOA1, STXBP6, SFRP1, PTCH1, THBS1, FN1* |
| GO:0051051 negative regulation of transport | *RHBDF2, SREBF1, CHGA, APOA1, SFRP1, KEL, RSAD2, THBS1, ADIPOQ, ADA, FN1* |
| GO:0051057 positive regulation of small GTPase mediated signal transduction | *APOA1, RASGEF1A, LPAR1* |
| GO:0051093 negative regulation of developmental process | *SFRP1, TNMD, IRF1, TBC1D7, PTCH1, LPAR1, GLI2, THBS1, ADIPOQ* |
| GO:0051094 positive regulation of developmental process | *P2RX7, APOA1, AGT, HSPB1, PTCH1, LOXL2, GLI2, IL7R, THBS1, ADIPOQ, ADA, FN1* |
| GO:0051224 negative regulation of protein transport | *RHBDF2, SREBF1, APOA1, SFRP1, RSAD2, ADIPOQ, FN1* |
| GO:0051239 regulation of multicellular organismal process | *SREBF1, CEBPB, KEL, PDK4, TNMD, PRRX1, RSAD2, LPAR1, IL7R, GLI2, ADIPOQ, ADA, OGN, P2RX7, APOA1, SFRP1, AGT, IRF1, HSPB1, PTCH1, THBS1, LOXL2, FN1* |
| GO:0051240 positive regulation of multicellular organismal process | *P2RX7, CEBPB, AGT, IRF1, HSPB1, RSAD2, LOXL2, GLI2, IL7R, THBS1, ADIPOQ, ADA, FN1* |
| GO:0051241 negative regulation of multicellular organismal process | *P2RX7, APOA1, SFRP1, TNMD, IRF1, PTCH1, LPAR1, GLI2, THBS1, ADIPOQ, ADA, FN1* |
| GO:0051592 response to calcium ion | *P2RX7, THBS1, ITPR3* |
| GO:0051674 localization of cell | *ITGA11, RASGEF1A, LPAR1, CD151, ADIPOQ, ADA, CHGA, APOA1, SFRP1, AGT, PDGFRA, HSPB1, JAK2, THBS1, LOXL2, FN1* |
| GO:0051707 response to other organism | *NLRC5, P2RX7, CHGA, CEBPB, AVD, PDK4, IRF1, HSPB1, RSAD2* |
| GO:0055080 cation homeostasis | *P2RX7, EPB42, KEL, PDK4, LPAR1, ITPR3, SLC26A2, RHAG* |
| GO:0055123 digestive system development | *SFRP1, PDGFRA, GLI2, ADA* |
| GO:0060021 palate development | *MEOX2, BNC2, PDGFRA, PRRX1* |
| GO:0060326 cell chemotaxis | *CHGA, PDGFRA, HSPB1, LPAR1, THBS1* |
| GO:0061053 somite development | *MEOX2, SFRP1, PTCH1, FN1* |
| GO:0061061 muscle structure development | *FAM65B, RBM24, MYH15, APOA1, MEOX2, MAPK12, KEL, PDGFRA, RBM38* |
| GO:0061138 morphogenesis of a branching epithelium | *SFRP1, AGT, PTCH1, GLI2* |
| GO:0061448 connective tissue development | *CYTL1, PRRX1, LOXL2, GLI2, CBS* |
| GO:0070208 protein heterotrimerization | *COL6A1, ADIPOQ* |
| GO:0070661 leukocyte proliferation | *P2RX7, CEBPB, IRF1, NPR3, IL7R, CD151, ADA* |
| GO:0070887 cellular response to chemical stimulus | *CREBRF, SREBF1, CRIP1, CEBPB, PDK4, LPAR1, COL16A1, GLI2, ADIPOQ, RASL11B, NLRC5, P2RX7, CHGA, APOA1, SFRP1, AGT, IRF1, PDGFRA, COL6A1, HSPB1, JAK2, PTCH1, THBS1* |
| GO:0071310 cellular response to organic substance | *CREBRF, SREBF1, CEBPB, PDK4, COL16A1, GLI2, ADIPOQ, RASL11B, NLRC5, P2RX7, APOA1, SFRP1, AGT, IRF1, PDGFRA, COL6A1, HSPB1, JAK2, PTCH1, THBS1* |
| GO:0071356 cellular response to tumor necrosis factor | *APOA1, SFRP1, JAK2, ADIPOQ* |
| GO:0071495 cellular response to endogenous stimulus | *RASL11B, SREBF1, CREBRF, CEBPB, SFRP1, PDK4, PDGFRA, COL6A1, JAK2, THBS1, COL16A1, ADIPOQ* |
| GO:0071496 cellular response to external stimulus | *SREBF1, P2RX7, SFRP1, PDK4, IRF1* |
| GO:0071593 lymphocyte aggregation | *P2RX7, CEBPB, IRF1, RSAD2, IL7R, CD151, ADA* |
| GO:0071900 regulation of protein serine/threonine kinase activity | *P2RX7, SFRP1, HSPB1, LPAR1, THBS1, ADIPOQ, CBS* |
| GO:0072358 cardiovascular system development | *MYH15, MEOX2, TNMD, PDGFRA, PRRX1, HSPB1, PTCH1, LOXL2, GLI2, COL8A1, THBS1, FN1* |
| GO:0072359 circulatory system development | *MYH15, MEOX2, TNMD, PDGFRA, PRRX1, HSPB1, PTCH1, LOXL2, GLI2, COL8A1, THBS1, FN1* |
| GO:0080134 regulation of response to stress | *NLRC5, APOA1, SFRP1, PDK4, IRF1, PRRX1, RSAD2, HSPB1, JAK2, THBS1, ADIPOQ, ADA, CBS* |
| GO:0090288 negative regulation of cellular response to growth factor stimulus | *RASL11B, SFRP1, AGT, THBS1* |
| GO:0098771 inorganic ion homeostasis | *P2RX7, EPB42, KEL, PDK4, LPAR1, ITPR3, SLC26A2, RHAG* |
| GO:1901698 response to nitrogen compound | *SREBF1, P2RX7, CEBPB, SFRP1, PDK4, PDGFRA, COL6A1, JAK2, COL16A1, ADIPOQ, CBS* |
| GO:1901699 cellular response to nitrogen compound | *SREBF1, P2RX7, CEBPB, SFRP1, PDK4, PDGFRA, COL6A1, JAK2, COL16A1, ADIPOQ* |
| GO:1901700 response to oxygen-containing compound | *SREBF1, P2RX7, CEBPB, SFRP1, PDK4, PDGFRA, COL6A1, JAK2, PTCH1, THBS1, COL16A1, ADIPOQ, CBS* |
| GO:1901701 cellular response to oxygen-containing compound | *SREBF1, CEBPB, SFRP1, PDK4, PDGFRA, COL6A1, PTCH1, JAK2, COL16A1, ADIPOQ* |
| GO:1902105 regulation of leukocyte differentiation | *CEBPB, SFRP1, IRF1, IL7R, ADIPOQ, ADA* |
| GO:1903530 regulation of secretion by cell | *RHBDF2, SREBF1, P2RX7, CHGA, APOA1, SFRP1, SLC6A2, RSAD2, FN1* |
| GO:1903706 regulation of hemopoiesis | *CEBPB, SFRP1, IRF1, IL7R, ADIPOQ, ADA* |
| GO:1904950 negative regulation of establishment of protein localization | *RHBDF2, SREBF1, APOA1, SFRP1, RSAD2, ADIPOQ, FN1* |
| GO:0097191 extrinsic apoptotic signaling pathway | *P2RX7, SFRP1, AGT, G0S2, THBS1* |
| GO:0098542 defense response to other organism | *NLRC5, P2RX7, CHGA, CEBPB, AVD, PDK4, IRF1, RSAD2* |
| GO:0098602 single organism cell adhesion | *CEBPB, RSAD2, CD151, IL7R, ADIPOQ, ADA, MYL9, P2RX7, APOA1, TENM2, IRF1, HSPB1, FN1, KIF26B* |
| GO:0098609 cell-cell adhesion | *P2RX7, APOA1, CEBPB, TENM2, IRF1, HSPB1, RSAD2, IL7R, CD151, ADIPOQ, ADA, MYL9, KIF26B* |
| GO:1990267 response to transition metal nanoparticle | *P2RX7, CRIP1, LOXL2* |
| GO:2000026 regulation of multicellular organismal development | *CEBPB, KEL, TNMD, PRRX1, LPAR1, IL7R, GLI2, ADIPOQ, ADA, OGN, P2RX7, APOA1, SFRP1, AGT, IRF1, HSPB1, PTCH1, THBS1, LOXL2, FN1* |
